# Multimodal CRISPR perturbations of GWAS loci associated with coronary artery disease in vascular endothelial cells

**DOI:** 10.1371/journal.pgen.1010680

**Published:** 2023-03-16

**Authors:** Florian Wünnemann, Thierry Fotsing Tadjo, Mélissa Beaudoin, Simon Lalonde, Ken Sin Lo, Benjamin P. Kleinstiver, Guillaume Lettre

**Affiliations:** 1 Montreal Heart Institute, Montréal, Québec, Canada; 2 Center for Genomic Medicine and Department of Pathology, Massachusetts General Hospital, Boston, Massachusetts, United States of America; 3 Department of Pathology, Harvard Medical School, Boston, Massachusetts, United States of America; 4 Faculté de Médecine, Université de Montréal, Montréal, Québec, Canada; Stanford University, UNITED STATES

## Abstract

Genome-wide association studies have identified >250 genetic variants associated with coronary artery disease (CAD), but the causal variants, genes and molecular mechanisms remain unknown at most loci. We performed pooled CRISPR screens to test the impact of sequences at or near CAD-associated genetic variants on vascular endothelial cell functions. Using CRISPR knockout, inhibition and activation, we targeted 1998 variants at 83 CAD loci to assess their effect on three adhesion proteins (E-selectin, ICAM1, VCAM1) and three key endothelial functions (nitric oxide and reactive oxygen species production, calcium signalling). At a false discovery rate ≤10%, we identified significant CRISPR perturbations near 42 variants located within 26 CAD loci. We used base editing to validate a putative causal variant in the promoter of the *FES* gene. Although a few of the loci include genes previously characterized in endothelial cells (*e*.*g*. *AIDA*, *ARHGEF26*, *ADAMTS7*), most are implicated in endothelial dysfunction for the first time. Detailed characterization of one of these new loci implicated the RNA helicase *DHX38* in vascular endothelial cell senescence. While promising, our results also highlighted several limitations in using CRISPR perturbations to functionally dissect GWAS loci, including an unknown false negative rate and potential off-target effects.

## Author summary

Genome-wide association study (GWAS) is a method designed to identify genetic variants that increase the risk to develop common human diseases such as heart attacks or schizophrenia. While powerful, this method has one major limitation: it cannot unambiguously pinpoint the genes responsible for the diseases. This important step requires investigators to experimentally test genes located near the variants identified by GWAS for functions related to the diseases of interest. Here, we used a technique called CRISPR to test if genes near genetic variants implicated by GWAS in heart attacks modulate the functions of vascular endothelial cells. Endothelial cells form the inner layer of blood vessels, and play a critical role in the development of the pathology (atherosclerosis) that leads to heart attacks. In total, we found 26 regions in the human genome that include heart attacks-associated variants and that influence the functions of endothelial cells. Focusing specifically on one of our findings, we showed that the gene *DHX38* regulates a process called senescence, which in turn modulates how endothelial cells respond to stimuli that promote heart attacks.

## Introduction

Coronary artery disease (CAD) remains the main cause of mortality in the world despite widely available drugs (*e*.*g*. statins) and the known benefits of simple prevention strategies (*e*.*g*. exercise). Part of the complexity to prevent and treat CAD resides in our incomplete understanding of atherosclerosis, the pathophysiological process largely responsible for CAD initiation and progression. Atherosclerosis is triggered by many environmental risk factors and other intrinsic stimuli, and results in the dysregulation of vascular wall homeostasis due to the accumulation of cholesterol-rich lipoproteins and a maladaptive inflammatory state [1,2].

Human genetics provides a framework to dissect the biological pathways and cellular networks implicated in atherosclerosis. Genome-wide association studies (GWAS) have already identified >250 loci associated with CAD [3–5]. However, the functional characterization of genes that modulate CAD risk at GWAS loci is labor-intensive. It is further complicated by the fact that most CAD variants are non-coding and are in linkage disequilibrium (LD) with a multitude of other DNA sequence variants.

Half of the CAD GWAS loci do not associate with traditional risk factors. We and others have hypothesized that some of the CAD variants, which are enriched in open chromatin regions found in human vascular endothelial cells, directly modulate endothelial cell functions [6,7]. The functional characterization of two CAD genes in endothelial cells, *PLPP3* [6] and *AIDA* [7], has further supported this hypothesis. Vascular endothelial cells have critical roles in atherosclerosis [8,9]. Upon activation, they express adhesion molecules necessary for monocyte rolling and attachment (*e*.*g*. E-selectin, ICAM1, VCAM1) and weakening of their cell-cell junctions can facilitate monocyte transmigration into the intima. Furthermore, dysfunctional endothelial cells adopt an atheroprone behaviour with changes in calcium (Ca^2+^) signalling [10], decreased bioavailability of the vasodilator nitric oxide (NO) and increased production of reactive oxygen species (ROS).

Dysfunctional endothelial cells can also undergo senescence, which is a stress response that results in stable cell cycle arrest [11]. It can be induced by different stimuli (*e*.*g*. from ROS) and senescent cells accumulate in aging tissues to impair normal functions. Senescent cells present with cell-to-cell phenotypic heterogeneity, including transcriptomic variability, that depends on the stress inducers and cell types [12,13]. Senescence has been divided between primary and secondary senescence [14]. Primary senescence occurs in cells in direct response to the stress and these cells can induce secondary senescence in the surrounding cells through paracrine signalling mediated by the secretion of inflammatory cytokines, growth factors and proteases, altogether termed the senescence-associated secretory phenotype (SASP) [15]. Senescent endothelial cells are characterized by a pro-inflammatory and atheroprone phenotype that involves increased production of adhesion molecules (*e*.*g*. E-selectin, ICAM1, VCAM1) and ROS, impaired Ca^2+^ signaling and reduced NO bioavailability [16]. To date, endothelial cell senescence has not been implicated as a potential pathological mechanism for CAD based on GWAS discoveries.

The development of pooled CRISPR-based screens now allows perturbation experiments to test sequences at or near most sentinel and LD proxy variants associated with CAD for a role in human vascular endothelial cells [17]. Moreover, by using inhibition (KRAB) or activation (VP64) domains tethered to an inactivated Cas9 (dCas9), it is possible to mimic loss- or gain-of-function effects that might elude perturbations due to classic Cas9 insertion-deletions (indels) [18–20]. Here, we carried out pooled CRISPR screens for six endothelial phenotypes relevant to atherosclerosis (presentation of adhesion proteins at the cell membrane (E-selectin, ICAM1 and VCAM1), production of NO and ROS, and intracellular Ca^2+^ concentration) using three different Cas9 perturbation modalities (double-strand break induction (Cas9), inhibition (dCas9-KRAB or CRISPRi) and activation (dCas9-VP64 or CRISPRa)). Through these experiments, we aimed to identify CAD-associated variants that modulate endothelial functions. More generally, we also evaluated whether pooled CRISPR screens are a robust and comprehensive methodology to characterize GWAS loci, especially when the likely causal variants are non-coding. Our results suggest that the method is useful to prioritize variants and genes, but requires additional experimental validation to rule out false positive and negative findings.

## Results

### FACS-based pooled CRISPR screens for endothelial functions

**Fig 1:**
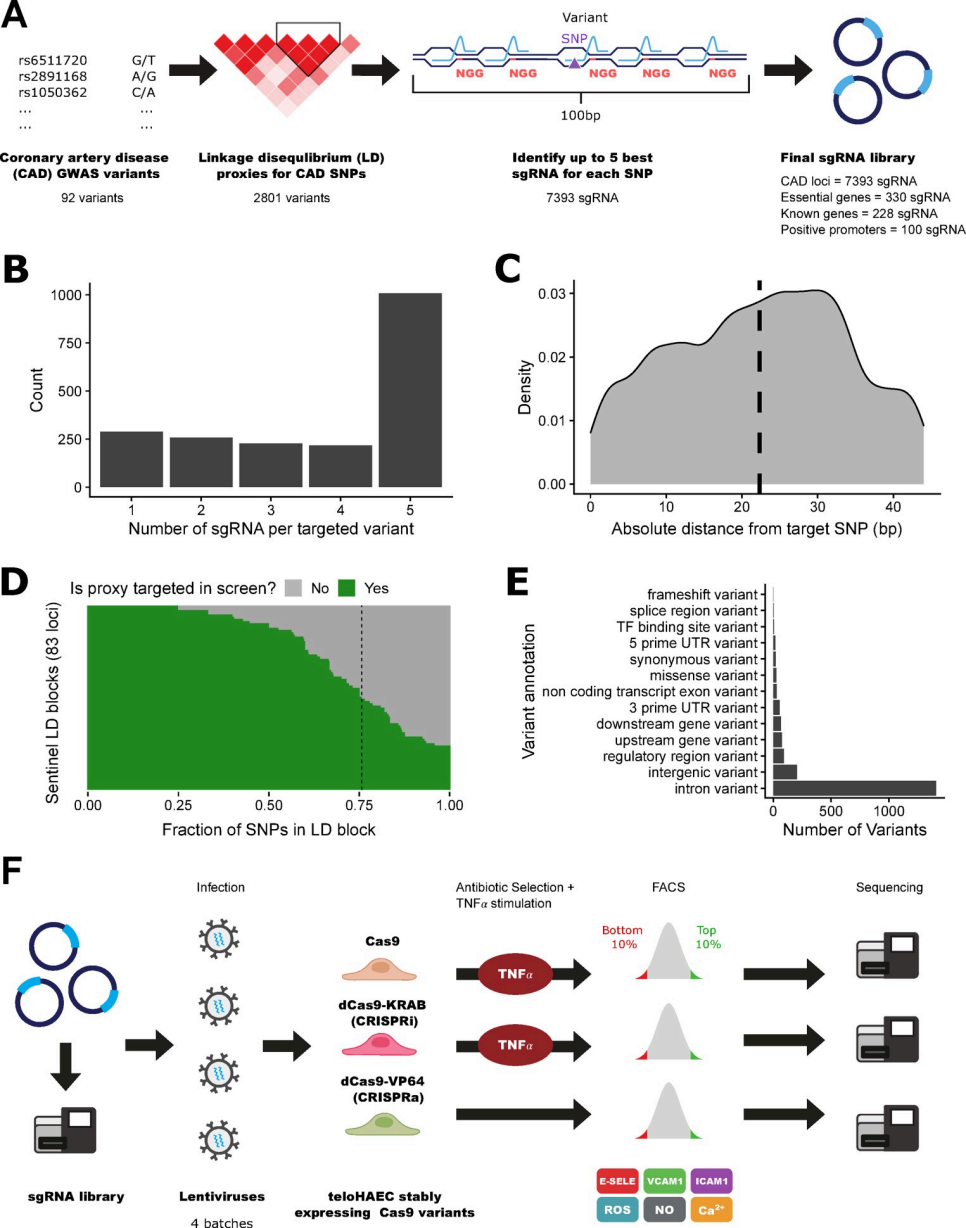
Pooled CRISPR screens to identify CAD variants and genes that modulate vascular endothelial functions. (**A**) From 92 loci associated with coronary artery disease (CAD) risk by genome-wide association studies (GWAS), we identified 2893 sentinel and linkage disequilibrium proxy variants for testing. For each of these variants, we attempted to design a maximum of five high-quality guide RNAs (sgRNAs) within a 100-bp window. In the design of the library, we also included sgRNAs that target genes essential for cell viability, as well as sgRNAs that target the coding sequence and promoter of genes that control endothelial cell functions (known genes, positive controls). (**B**) Number of sgRNAs per targeted variant that passed stringent quality-control filters. In total, we designed 7393 sgRNAs against 1998 CAD-associated variants (mean and median number of sgRNA per variant are 3.7 and 5, respectively). (**C**) Distribution of the absolute distance of the sgRNA cut-site relative to the targeted variant in base pairs (the vertical dashed line indicates mean sgRNA distance). (**D**) Fraction of variants at each locus that are successfully targeted by our pooled CRISPR screens. Each row represents one of the CAD loci that we tested. In green is the fraction of variants—including sentinel and LD proxies—for which we designed high-quality sgRNAs and obtained results for the endothelial function phenotypes. On average, 76% of variants at any given CAD locus are captured in the screens (vertical dashed line). (**E**) Most severe annotation for the 1998 CAD variants targeted by the lentiviral sgRNA libraries using ENSEMBL’s Variant Effect Predictor (VEP) module. (**F**) As a control step, we sequenced the plasmid library to ensure even representation of sgRNAs in the pool. Then, we produced four independent batches of lentiviruses which we used to infect teloHAEC cells that stably express Cas9, dCas9-KRAB (CRISPRi) or dCas9-VP64 (CRISPRa). Following antibiotic selection and TNFα treatment (for Cas9 and CRISPRi), we stained teloHAEC for cell surface markers (E-selectin, ICAM-1, VCAM-1) or intracellular signaling molecules (reactive oxygen species (ROS), nitric oxide (NO), calcium (Ca^2+^)). By flow cytometry, we sorted cells from the bottom and top 10 percentiles of the marker distributions, and sequenced sgRNAs found in each fraction.

The design of our sgRNA library is summarized in **Fig 1A**. To target genomic regions associated with CAD, we collected 92 GWAS sentinel variants at 89 CAD-associated loci [21–24] and retrieved their proxy variants in strong LD (*r*^2^ >0.8 in populations of European ancestry). Using this strategy, we derived a set of 2,893 variants (92 GWAS sentinel and 2,801 LD proxy variants) (**Fig 1A** and **S1 Table**). For each of these variants, we designed a maximum of five high-quality sgRNAs (**Fig 1B**). The mean distance between sgRNA potential cut-sites and the targeted variants was 22-bp based on available PAM sites (**Fig 1C**). Using FORECAST [25], we estimated that 10±16% of the CRISPR/Cas9-mediated deletion alleles would disrupt the targeted variants (**S1 Fig**). After quality-control steps, we generated a list of 7,393 sgRNA that targeted sequences at or near 1,998 variants at 83 CAD loci (**S2 Table**). On average at each CAD locus, our sgRNA library covered 76±22% of the targeted variants (**Fig 1D** and **S3 Table**). Of the 83 tested loci, we could capture 100% of the targeted variants at 20 CAD loci and ≥80% of variants at 38 loci (**S3 Table**). The majority of the targeted variants are in intronic (70.8%) or intergenic (10.2%) sequences, 7.7% of the variants overlap with ATAC-seq peaks identified in immortalized human aortic endothelial cells (teloHAEC), and 4.1% are located in predicted enhancers identified in human primary endothelial cells and connected to target genes by the EpiMap Project (**Fig 1E** and **S4 Table**) [7,26]. Even if most targeted variants are non-coding, we decided to include Cas9 as a perturbation modality in our screens because we reasoned that indels within or near regulatory sequences (*e*.*g*. transcription factor motif) could impact cellular phenotypes [27–29].

**Fig 2:**
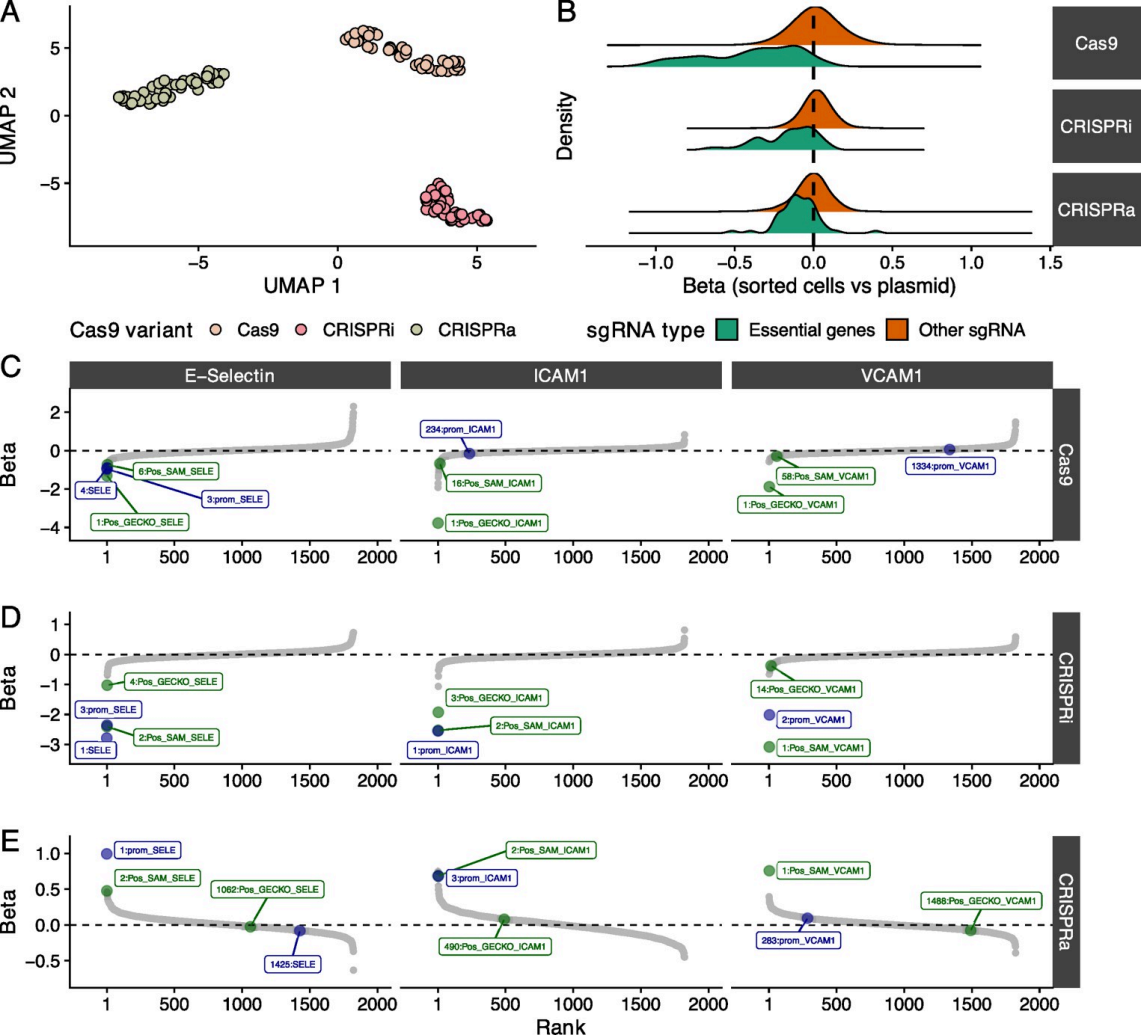
Quality-controls of the pooled CRISPR screens for vascular endothelial cell phenotypes. (**A**) Two-dimensional uniform manifold approximation and projection (UMAP) representation of 148 fluorescence-activated cell sorting (FACS) samples based on the normalized read counts of the top 10% most variable sgRNAs across all samples. (**B**) Density distributions of effect sizes (Beta, *x*-axis) across all Cas9 variants for essential genes and the rest of the sgRNA library. Positive betas indicate that sgRNA are enriched in the cell fractions when compared to the input library, while negative betas indicate a depletion of sgRNA across all samples. We observed a depletion of sgRNA targeting essential genes with all three Cas9 variants. (**C-E**) Rank of all control sgRNAs and targeted CAD variants in the (**C**) Cas9, (**D**) CRISPRi and (**E**) CRISPRa screens for three adhesion proteins: E-selectin (left), VCAM1 (middle) and ICAM1 (right). For each panel, the *y*-axis corresponds to the effect sizes (Beta, comparing top *vs* bottom FACS 10% fractions). For the Cas9 and CRISPRi experiments, we found an enrichment of sgRNAs targeting the coding and promoter sequences of genes encoding adhesion proteins in the bottom 10% cell fractions (negative Betas). In contrast, sgRNAs targeting the promoter of these genes were enriched in the top 10% cell fractions in the CRISPRa experiments. In green and blue, we highlight sgRNAs targeting coding exons and promoters, respectively. The number in front of the name of each control sgRNA indicates its rank in the corresponding analysis.

We utilized lentiviruses to deliver our pooled CRISPR libraries to teloHAEC that stably express one of three Cas9 variants (Cas9, CRISPRi, CRISPRa) (**Fig 1F**). We treated Cas9 and CRISPRi (but not CRISPRa) infected cells with TNFα in order to find genes that can block (Cas9, CRISPRi) or induce (CRISPRa) a pro-inflammatory response. We labelled cells with fluorescent antibodies against E-selectin, VCAM1, or ICAM1, or with fluorescent dyes for signalling molecules (ROS, NO, Ca^2+^), and sorted cell populations by flow cytometry (FACS) to collect the bottom and top 10% cells based on fluorescence intensity (**Figs 1F** and **S2–S4**). We amplified and sequenced the sgRNAs from the FACS cell fractions to identify sgRNAs that have a significant effect on endothelial functions. For each screen, we performed and combined results from at least four independent biological replicates (sgRNA-level correlation analyses between replicates are in **S5 Table**). Quality-control analyses of sorted cell fractions showed a good representation of sgRNA diversity (mean Gini index = 0.076±0.01) and a good read coverage per sgRNA (mean number of aligned reads per sgRNA = 1995±2981) (**S5 Fig**). Analysis of the 10% most variable sgRNAs across all assays revealed clustering of samples along the Cas9 modalities (**Fig 2A**).

### Effects of CRISPR knockout, inhibition and activation in teloHAEC

To assess Cas9 efficiency in our experiments, we included in the library 330 sgRNAs against the coding sequence of genes essential for cell viability. For Cas9 and CRISPRi, we found a strong depletion of sgRNAs targeting essential genes among the sequenced FACS cell fractions (Kolmogorov-Smirnov (KS) test *P*<2.2x10^-16^ and *P* = 1.6x10^-13^, respectively) (**Fig 2B**). We also noted a minor but significant shift toward depletion in the sgRNA count distribution of essential genes for the CRISPRa experiments (KS test *P* = 3.7x10^-6^), potentially due to steric hindrance effects by the dead Cas9 moiety near the transcriptional start site of these genes or the toxic impact of gene over-expression (**Fig 2B**).

As an additional quality-control step in our experiment, we designed sgRNAs against the coding and promoter sequences of *SELE*, *ICAM1* and *VCAM1*, which encode the three adhesion proteins measured in our FACS assays (**Fig 1F**). We observed significant depletion of sgRNAs targeting coding exons and promoter regions of these genes in the top vs. bottom 10% FACS fractions with Cas9 or CRISPRi (**Figs 2C and 2D and S6**). In the CRISPRa experiments, the same sgRNAs were enriched in FACS fractions with high E-selectin, ICAM1 or VCAM1 levels (**Figs 2E** and S6). The three other endothelial phenotypes measured in our experiments—NO and ROS production, and Ca^2+^ signalling—are physiological readouts that are not the product of a single gene. In the absence of confirmed positive control genes that we could target to validate our system, we carefully calibrated the flow cytometry assays for these readouts using appropriate agonists/inducers. Our screens are sufficiently sensitive to detect sgRNAs targeting CAD loci that have strong effects on these hallmarks of endothelial dysfunction. It is also important to mention that CRISPR perturbation screens will miss variants or genes that cause small phenotypic effects, for instance because of gene redundancy or cellular compensation, or because of an impact on cell proliferation.

**Fig 3:**
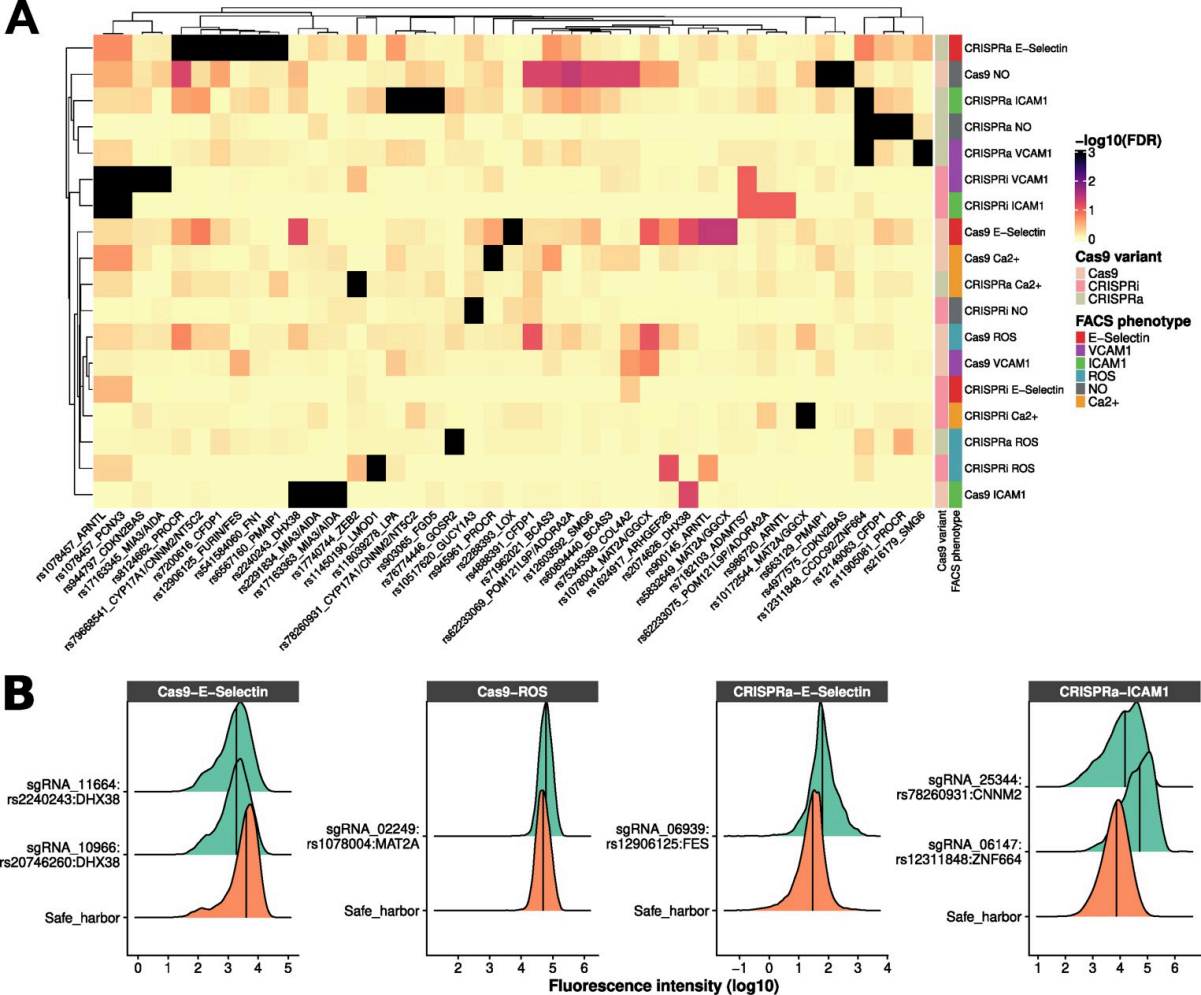
Discovery and validation of CRISPR perturbations that induce atheroprone vascular endothelial cell phenotypes. (**A**) Heatmap of CAD-associated variants that are significant (false discovery rate (FDR) ≤10%) for at least one of six endothelial phenotypes tested in the teloHAEC pooled CRISPR screens. Each row corresponds to a combination of Cas9 variant and cellular readout, and each column corresponds to a CAD variant. For each variant, we added the name of a nearby gene to simplify locus identification, although we do not imply that these genes are causal. Dendrograms of rows and columns represent hierarchical clustering based on euclidean distance. The FDR is capped at 0.1%. (**B**) Validation by flow cytometry of six hits from the pooled CRISPR screens. For each validation, we used the top sgRNA from the pooled CRISPR screens to target the variant/locus with the corresponding Cas9 variant. We compared the distribution of the fluorescence intensity of the cellular markers (*x*-axis) between the sgRNA identified in the screens and a safe harbor negative control sgRNA. We assessed statistical significance using the Kolmogorov-Smirnov (KS) test, all validations shown are significant (KS P-value <2.2x10^-16^). Validations were performed in at least three independent experiments for each sgRNA (**S6 Table**). For E-selectin and ICAM1, the fluorochrome is PE; for ROS, the fluorochrome is FITC.

Our pooled CRISPR screens compared sgRNA frequencies between the bottom and top 10% fractions for each cellular readout. Using MAGeCK’s maximum likelihood estimation method that combines results for sgRNA that target the same variant (**Methods**), we identified 51 significant variant-endothelial phenotype results (false discovery rate (FDR) ≤10%) involving 42 different variants located within 26 CAD loci (**Fig 3A** and **S4 Table**). The majority of these 42 variants is located in non-coding regions: 30 variants are in introns, 5 variants are in intergenic regions, four variants are in exons and three variants are in promoters (**S4 Table**). The 42 variants were also not enriched in ATACseq peaks identified in teloHAEC (9.5% (4/42) vs. 7.7% for all targeted variants in the screens; binomial *P* = 0.56). We found significant results for almost all combinations of Cas9 modality and FACS phenotypes, and most of these results were specific to a single combination (**Fig 3A**). For 15 CAD loci where we could target all LD proxies with sgRNAs (**Fig 1D** and **S3 Table**), we detected no significant signals in our CRISPR assays, suggesting that genes within these genomic regions modulate CAD risk through different functions or cell types, or that our functional assays were not sensitive enough to capture their effects. When compared with genomic loci with no significant results, CAD loci with at least one significant variant in our CRISPR screens were not better captured by designed sgRNAs (median coverage 73% vs. 78% of LD proxies, Wilcoxon’s test *P* = 0.70) but had significantly more LD proxies (median 41 vs. 9 variants, Wilcoxon’s test *P* = 1.1x10^-4^).

Several of the CAD loci identified by GWAS have been implicated in blood lipid metabolism (*e*.*g*. *LDLR*, *APOE*, *PCSK9*). Because genetic variation within these loci are likely to influence risk through an effect on lipid levels, we did not anticipate to identify them in our endothelial cell functions CRISPR screens. Of the variants that mapped to 10 lipid loci included in our screens, all were negative across the different endothelial phenotypes tested except rs118039278 located in an intron of *LPA* (CRISPRa for ICAM1, FDR<0.001, **Fig 3A**). Although *LPA* is not expressed in teloHAEC, CRISPRa could induce its ectopic expression and the encoded Lp(a) lipoprotein has been shown to induce endothelial dysfunction [30].

### Validation using single sgRNA experiments

To validate our results, we selected eight CRISPR perturbations at seven CAD loci and performed individual sgRNA infection and FACS experiments (**S6 Table**). For this validation step, we prioritized variants that were significant for >1 cellular phenotypes and that had strong effect sizes in the CRISPR screens. For each experiment, we compared the distribution of the FACS-based cellular phenotype between control sgRNAs and the best sgRNA targeting each selected CAD variant (**Fig 3B**). Across three independent biological replicates, we could validate six of the eight selected CRISPR perturbations (one-tailed *t*-test *P*<0.05, **S6 Table**). One of the replicated sgRNA implicated an outstanding candidate CAD gene: for *MAT2A*, targeting Cas9 to the synonymous rs1078004 variant increased ROS production in TNFα-treated teloHAEC (**Fig 3B**). *MAT2A* encodes a methionine adenosyltransferase that is responsible for the biosynthesis of S-adenosylmethionine, a precursor of the potent antioxidant glutathione [31].

### Base editing validates a putative functional CAD variant near *FES*

**Fig 4:**
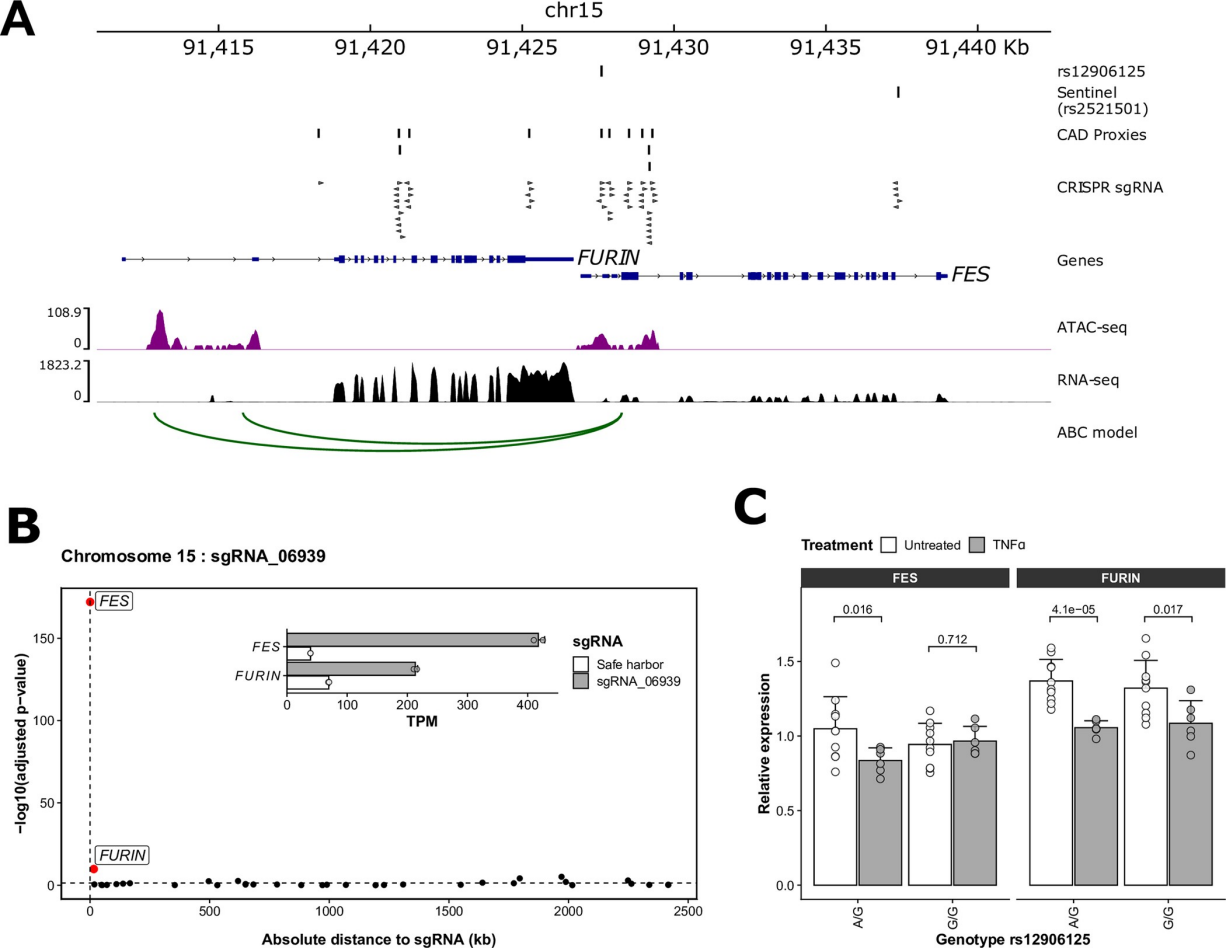
Characterization of a CAD-associated regulatory variant located within an enhancer at the *FURIN*/*FES* locus. (**A**) CRISPRa perturbations highlighted rs12906125 as a potential regulatory variant for *FURIN* and *FES*. The variant overlaps an ATAC-seq peak in the promoter of *FES* and a H3K27ac-defined enhancer that physically interacts with the *FURIN* promoter through chromosomal loops predicted by the ABC model applied to teloHAEC Hi-C data [7,33]. (**B**) Within a 2.5-Mb window, *FES* and *FURIN* are the top two differentially expressed genes when targeting rs12906125 by CRISPRa in teloHAEC. The inset plot shows the induction of both *FES* and *FURIN* expression with sgRNA_06939 when compared to the control safe harbor sgRNA. (**C**) teloHAEC are heterozygous (A/G) at rs12906125. We used base editing to change the genotype at rs12906125 to G/G. There was no significant difference in expression for *FES* and *FURIN* when comparing unstimulated teloHAEC with the A/G and G/G genotypes. However, upon activation with TNFα, we found that the reduction in *FURIN* levels was independent from the rs12906125 genotype whereas for *FES*, the reduction was genotype-dependent. Numbers above the bars are Student’s *t*-test *P*-values. We tested at least six clones of each genotype.

A CRISPRa/E-selectin perturbation that we replicated implicated rs12906125, a variant at the *FURIN/FES* locus previously prioritized as potentially causal for CAD by transcriptomic and epigenomic profiling in human endothelial cells [32]. The corresponding Cas9 knockout and CRISPRi results for E-selectin were non-significant, suggesting that gene activation is required to reveal an endothelial phenotype as this locus (**S4 Table**). rs12906125 is in strong LD with the CAD sentinel variant rs2521501 (*r*^2^ = 0.91), is located in the *FES* promoter and overlaps an ATAC-seq peak as well as a H3K27ac-defined enhancer that physically interacts with the *FURIN* promoter (**Fig 4A**) [7]. The same SNP is an eQTL for *FES* in human primary aortic endothelial cells [32] and arterial tissues from GTEx.

In the CRISPRa experiment using RNA-seq, we found a significant up-regulation of both *FES* (log_2_(fold-change (FC)) = 3.75, adjusted *P* = 8.5x10^-173^, rank_cis_ = 1, rank_trans_ = 4) and *FURIN* (log_2_FC = 0.78, adjusted *P* = 1.5x10^-10^, rank_cis_ = 2, rank_trans_ = 197) (**Fig 4B**). *FURIN*, which encodes a proprotein convertase, represents a strong candidate CAD causal gene at this locus: its specific knockdown in human endothelial cells reduces atheroprone characteristics such as monocyte-endothelial adhesion and transmigration [34]. In contrast, *FES*, which encodes a tyrosine protein kinase that can control cell growth, differentiation and adhesion, has not been implicated in vascular endothelial cell biology. To determine which of the two is the more likely causal CAD gene, we changed the genotype at rs12906125 in teloHAEC using adenosine base editing. teloHAEC are heterozygous at rs12906125 (A/G) and we could generate clones with the G/G genotype (because of the sequence context, we could not get A/A teloHAEC cells with cytosine base editors directing C>T edits on the other strand). In unstimulated cells, genotype at rs12906125 had no impact on the expression of *FURIN* and *FES* (**Fig 4C**). However, because rs12906125 maps in the middle of an ATAC-seq peak and a binding motif for NFκB/p65 [32], we reasoned that the genotypic effect could be revealed by an inflammatory stimulus. Indeed, TNFα treatment reduced the expression of *FURIN* in teloHAEC with the A/G and G/G genotypes, whereas its effect on *FES* expression was genotype-dependent (**Fig 4C**). When we compared E-selectin expression at the mRNA and protein levels between A/G and G/G clones, the difference was not significant, potentially because we could not generate A/A clones and the effect of the A/G-to-G/G base edit on *FES* expression was modest in comparison to the CRISPRa impact (**S7 Fig**). While we cannot exclude *FURIN* as an excellent candidate causal CAD gene, our base editing experiment suggests that there might be >1 causal genes at this locus and that *FES* should be tested in future experiments aimed at determining its precise biological function(s) in atherosclerosis.

### Loss of *DHX38* function induces vascular endothelial cell senescence

**Fig 5:**
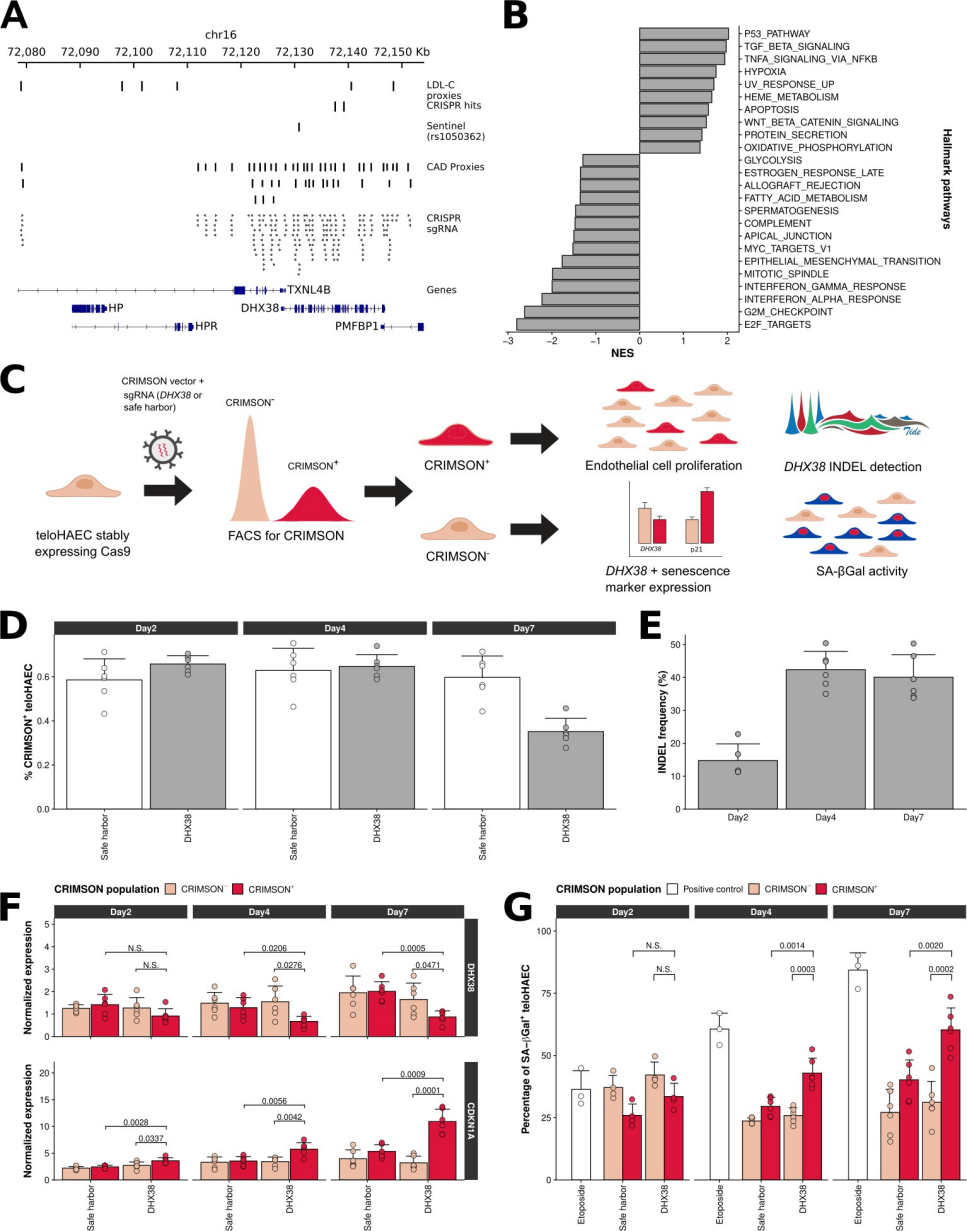
Disruption of *DHX38* induces vascular endothelial cell senescence. (**A**) Perturbations with the Cas9 nuclease highlighted two synonymous variants (rs2074626, rs2240243) in the *DHX38* gene for several endothelial phenotypes. *DHX38* is located downstream of the HP and HPR genes, which have previously been associated with LDL-C levels. However, the CAD and LDL-C GWAS signals are distinct based on co-localization analyses (posterior probability for two independent association signals (H3) = 80.9%). (**B**) Gene-set enrichment analysis results for differentially expressed genes identified by RNA-seq in teloHAEC between a sgRNA targeting a *DHX38* coding exon and a safe harbor negative control sgRNA. Only pathways with a Benjamini-Hochberg-corrected P-value <0.05 and normalized enrichment scores (NES) <-1 or >1 are shown. (**C**) Experimental design for the characterization of *DHX38* using the fluorescent marker CRIMSON in place of an antibiotic resistance gene. We did all experiments in teloHAEC that stably express Cas9. We monitored the impact of a *DHX38* sgRNA on cell proliferation, indel induction, gene expression and senescence-associated β-galactosidase (SA-βGal) activity. (**D**) Comparison of endothelial cell proliferation between teloHAEC with a *DHX38* sgRNA or a safe harbor negative control sgRNA. The differences in the number of CRIMSON^+^ cells were not significant two or four days post-infection. However, there were 27% less CRIMSON^+^ cells with *DHX38* sgRNA relative to the safe harbor control at seven days post-infection (Student’s *t*-test *P*-value = 7.3x10^-8^). Results are mean ± standard deviation for 6 replicates for safe harbor and three replicates for two DHX38 targeting sgRNA. (**E**) Quantification of *DHX38* indels by tracking of indel by decomposition (TIDE) analysis. As expected, we found no indels in the CRIMSON^-^ cells (**S8 Table**). However, in CRIMSON^+^ cells that received a *DHX38* sgRNA, we found indels with an average frequency of 15%, 42% and 40% at day 2, 4 and 7, respectively. Results are mean ± standard deviation for 6 replicates for safe harbor and three replicates for two DHX38 targeting sgRNA. (**F**) Expression levels of *DHX38* and *CDKN1A* in CRIMSON^-^ and CRIMSON^+^ teloHAEC that have received a sgRNA that targets *DHX38* or a safe harbor region (negative control). There were no significant differences in *DHX38* expression levels at day 2. However, at day 4 and 7, *DHX38* was significantly down-regulated and *CDKN1A* was significantly up-regulated in CRIMSON^+^ cells that received the *DHX38* sgRNA. N.S., not significant. We provide Student’s *t*-test P-values when *P*<0.05. Bars are mean normalized expression and error bars represent one standard deviation. (**G**) Quantification of senescent teloHAEC by flow cytometry using senescence-associated β-galactosidase (SA-βGal) staining. At day 4 and 7 post-infection, there were significantly more senescent cells in the CRIMSON^+^ *DHX38* sgRNA experiment than in the CRIMSON^-^ cells or in the CRIMSON^+^ cells that received the safe harbor sgRNA. We used the DNA damaging agent etoposide as a positive control to induce senescence. N.S., not significant. We provide Student’s *t*-test P-values when *P*<0.05. Results are mean percentage SA-βGal^+^ teloHAEC and error bars represent one standard deviation.

Two of the validated sgRNAs target exonic variants (rs2074626, rs2240243) in *DHX38* (**Fig 5A**). This GWAS CAD signal is located near an association signal for LDL-cholesterol (LDL-C), but a co-localization analysis suggests that the two signals are likely distinct (**Fig 5A**, coloc posterior probabilities H3:80.9% and H4:19.1%). Nonetheless, we cannot rule out the possibility that the *DHX38* variants may also contribute partially to CAD through an effect on LDL-C. *DHX38* encodes an RNA helicase involved in splicing, and mediate Cas9 nuclease effects on E-selectin (**Fig 3A and 3B**) and VCAM1 (as validated by subsequent analyses, **S6 Table**). We confirmed the *DHX38*-related E-selectin result using Cas9 ribonucleoprotein complexes (**S8 Fig**). In RNA-seq experiments with a sgRNA targeting *DHX38* (seven days post-infection, TNFα treatment), *DHX38* was not down-regulated and we found few reads mapping to *DHX38* with Cas9-mediated indels (<2%). However, we noted a strong gene expression signature suggesting an effect on cell proliferation with the modulation of genes involved in the p53, G2/M checkpoint and E2F target genes pathways (**Fig 5B** and **S7 Table**). To reconcile these observations, we hypothesized that endothelial cells with *DHX38* detrimental indels undergo senescence-mediated cell cycle arrest, have a growth disadvantage and induce a response in surrounding cells without *DHX38* indels through the SASP in a TNFα-stimulated environment.

To test this hypothesis, we replaced the antibiotic resistance marker by a fluorescence protein (CRIMSON) in the sgRNA vector in order to sort and characterize at different timepoints teloHAEC stably expressing Cas9 that have or not received a *DHX38* sgRNA (**Fig 5C**). While the fraction of CRIMSON^+^ cells is similar for safe harbor and *DHX38* sgRNAs two- and four-days post-infection, it is significantly lower after seven days (**Fig 5D**). Although we did not capture many *DHX38* indels in the RNA-seq experiment, we could detect a high frequency of indels (15–40%) in CRIMSON^+^ cells already two days post-infection (**Fig 5E** and **S8 Table**). Importantly, we also measured a down-regulation of *DHX38* expression levels in CRIMSON^+^ cells (**Fig 5F**).

In CRIMSON^+^ cells with *DHX38* sgRNA, we measured an up-regulation of *CDKN1A* (encoding the CDK2 inhibitor p21^WAF1/Cip1^) and detected a higher number of cells with β-galactosidase activity when compared to CRIMSON^-^ cells or CRIMSON^+^ cells with a safe harbor sgRNA (**Fig 5F and 5G**). These characteristics are hallmarks of cell senescence. We validated the effect of these sgRNAs on *DHX38* and *CDKN1A* expression levels using Cas9 ribonucleoprotein complexes in primary human coronary artery endothelial cells (**S9 Fig**). Activation of the senescence program is specific to *DHX38* and not a general response to DNA damage induced by this particular sgRNA as four different sgRNAs targeting two different *DHX38* exons impaired endothelial functions in the CRISPR screens.

### Two validated CRISPRa perturbations do not yield candidate CAD genes

Beside the *FURIN*/*FES* locus described above, we replicated two other CRISPRa perturbations that targeted intronic variants in *CNNM2* (rs78260931) and *ZNF664* (rs12311848) (**Fig 3A and 3B**). We used RNA-seq experiments to identify genes up-regulated near these variants that could mediate the CRISPRa effects on endothelial functions. While it has been reported that CRISPRa can lead to non-specific transcriptional effects such as the up-regulation of *IL6* [35], we used safe harbor sgRNAs to control for such effects, *IL6* was not differentially expressed in our experiments, and additional controls suggested a certain specificity of our CRISPRa results (**S10 Fig**).

Analysis of the RNA-seq data for the CRISPRa experiment at the *CNNM2*-rs78260931 locus revealed no evidence of differential expression for nearby genes (in *cis*, the closest differentially expressed gene was *NFKB2* located 568 kb away (log_2_FC = 0.33, adjusted *P* = 0.008)). We also manually inspected the sequence reads that mapped to the *CNNM2* region but did not find un-annotated genes that were differentially expressed (**Fig 3A**). Thus, based on our results, we cannot prioritize a candidate causal gene at this CAD locus. While this CRISPR signal could be a false positive finding due to an off-target effect, it is worth noting that the result is specific to this region and not a sgRNA-specific artifact because three of the four sgRNAs that we targeted at rs78260931 gave consistent results in the CRISPRa-ICAM1 screen (**S4 Table**).

**Fig 6:**
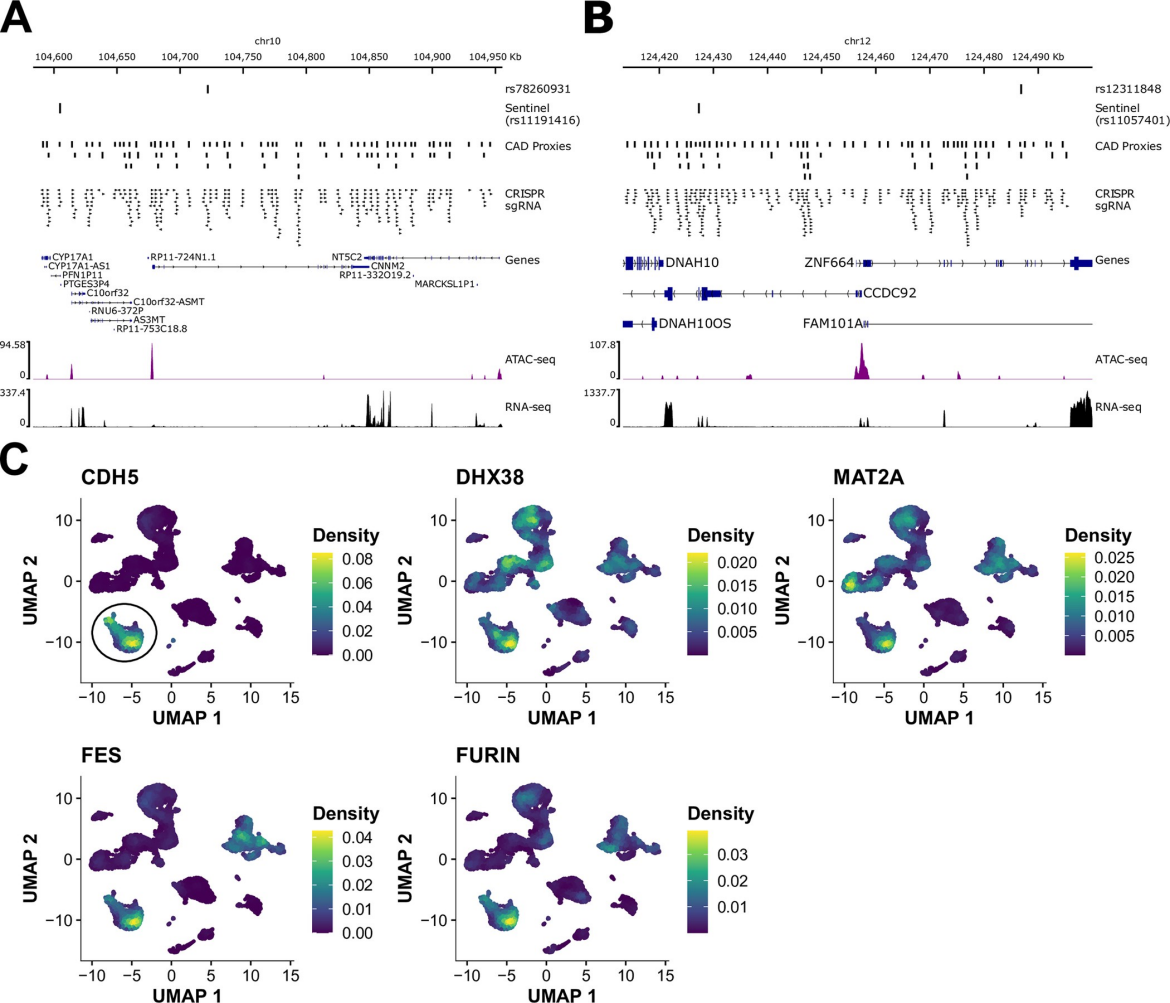
Validated CRISPRa effect at the *CNNM2* and *CCDC92*/*ZNF664* loci do not nominate candidate causal CAD genes. (A) Locus view for the CAD locus with nearby gene *CNNM2*. We provide the position of the sentinel CAD variant (rs11191416) and the putative functional variant identified in the pooled CRISPR screen (rs78260931). The LD proxies and sgRNAs tested are also shown. ATAC-seq and RNA-seq data in resting teloHAEC are from ref. [7]. (**B**) Locus view for the CAD locus with nearby genes *ZNF664* and *CCDC92*. We provide the position of the sentinel CAD variant (rs11057401) and the functional variant identified in the pooled CRISPR screen (rs12311848). The LD proxies and sgRNAs tested are also shown. ATAC-seq and RNA-seq data in resting teloHAEC are from ref. [7]. (**C**) Uniform manifold approximation projection (UMAP) for 11,756 cells from human right coronary arteries analyzed by single-cell RNA-sequencing [36]. We color-coded cells based on the level of expression of candidate causal CAD genes identified and characterized in this study. We used the expression of the endothelial cell marker gene *CDH5* (encoding VE-Cadherin) to identify endothelial cells (circle in top left panel). All five candidate genes are expressed in human vascular endothelial cells from coronary arteries.

Targeting CRISPRa at rs12311848 did not increase the expression of *ZNF664* but the expression of *CCDC92*, a gene located 29 kb upstream (log_2_FC = 0.74, adjusted *P* = 9.2x10^-5^, rank_cis_ = 3, rank_trans_ = 696, **Fig 6B**). The sentinel CAD variant identified by GWAS at this locus is rs11057401, a missense variant in *CCDC92*. We targeted four sgRNAs at rs11057401 but did not detect significant effects in the Cas9 nuclease nor CRISPRi screens. This result suggests that CRISPRa gain-of-function experiments are necessary to detect the impact of this locus on endothelial dysfunction. To support this hypothesis, we ectopically over-express the main *CCDC92* isoform in teloHAEC. While we measured a strong induction in *CCDC92* levels, we could not detect a significant change in the expression of *ICAM1*, the corresponding endothelial phenotype identified in the CRISPRa screen (**S11 Fig**). Therefore, either *CCDC92* is not the causal CAD gene at this locus, ectopic over-expression of the main *CCDC92* isoform is not sufficient to mimic the CRISPRa effect, or the screen result is spurious.

Finally, to determine if some of the candidate genes identified in this study could exert an effect in vascular endothelial cells *in vivo*, we re-analyzed single-cell RNA sequencing data from human coronary arteries [36]. We used the endothelial marker gene *CDH5* to identify a cluster of endothelial cells and confirmed that these cells express *DHX38*, *MAT2A*, *CCDC92*, *FES* and *FURIN*, prompting future efforts to dissect the role of these genes in this cell type in atherosclerosis (**Fig 6C**).

## Discussion

As for most complex human diseases, many GWAS loci associated with CAD do not include obvious candidate causal genes nor implicate known pathophysiological mechanisms. To elucidate their mechanisms and gain insights into atherosclerosis, we carried out multiple CRISPR screens to test if CAD variants impact vascular endothelial functions. By combining six different endothelial cell readouts and three Cas9 modalities, we identified sequences at or near 42 variants at 26 CAD loci (**Fig 3A**). This list is depleted of variants that modulate CAD risk through an effect on lipid metabolism and enriched for loci of unknown functions (**S3 Table**). We found sequences near *ARHGEF26*, *ADAMTS7*, and *GUCY1A3*, genes previously implicated in leukocyte transendothelial migration [23], endothelial cell angiogenesis [37], and NO signaling [38], respectively. We also retrieved rs17163363, an intronic variant in *MIA3* that controls the expression of *AIDA* in endothelial cells [7]. To document the false positive rate of our results, we tested eight sgRNAs prioritized in our screens and could validate six of them using the same FACS-based endothelial function readouts.

There were also variants and genes that we expected to find but did not recover. For instance, we did not identify rs17114036, a likely functional variant that controls the expression of *PLPP3* in endothelial cells, although this negative result may arise because the underlying enhancer requires hemodynamic stress to be active [6]. Furthermore, our screens did not yield variants at CAD loci that include *PECAM1* (adhesion protein CD31) and *NOS3* (endothelial NO synthase), two genes with important roles in endothelial cells. As for *PLPP3*, it might be that we did not activate endothelial cells with the right stimulus to detect the functional impact of these variants/genes in our assays. It is also possible that some loci will require the precise engineering of alleles (*e*.*g*. using base or prime editing) to detect a cellular phenotype, or that the phenotypic effect of a variant at the cellular level is too low to distinguish a true signal from the experimental noise inherent to any large-scale omics approach. One lesson learned from our experiments is that the false negative rate of such pooled CRISPR screens is likely not negligeable, implying that variants or genes should not be ruled out simply based on a non-significant CRISPR perturbation result.

We designed our sgRNA library using a variant-focused approach. However, it is likely that some of the findings from our CRISPR screens result from loss- or gain-of-function effects on endothelial genes independently of the causal variants. For instance, we identified and validated sgRNAs near synonymous variants in *DHX38* and *MAT2A* using the Cas9 nuclease. While synonymous variants can have phenotypic consequences, it is more likely that these variants are in LD with the causal variants but were captured in our screens because they targeted loss-of-function indels to the *DHX38* and *MAT2A* coding sequences. Similarly, ectopic activation or inhibition of gene expression by CRISPRa and CRISPRi can highlight candidate endothelial genes even if the sgRNAs do not directly overlap causal variants.

The main finding of our CRISPR experiments is the identification of *DHX38* as a strong candidate causal gene for CAD. Through Cas9-mediated deletions, we found that loss of *DHX38* functions in endothelial cells impairs cell cycle progression, induces the expression of the cell cycle inhibitor *CDKN1A*, and increases β-galactosidase activity, all hallmarks of cellular senescence. *DHX38*, also known as *PRP16*, encodes an RNA helicase implicated in splicing with previously described functions in tumorigenesis [39] and retina degeneration [40]. Interestingly, deregulation of RNA splicing through aging has been proposed as one mechanism leading to aging-related chronic diseases through an effect on cellular senescence [41]. While our results suggest that *DHX38* influences CAD risk by modulating endothelial dysfunction and senescence, it is possible that part of the CAD association signal at the locus is also due to nearby variants in weak linkage disequilibrium that associate with LDL-C (**Fig 5A**).

Some of the genes prioritized in our CRISPR perturbation screens (*e*.*g*. *FES*, *DHX38*) likely play essential cellular functions in endothelial cells. This could raise concerns about the specificity of some of our results and their relevance for CAD *in vivo*. But there is an alternative scenario in which the disruption of essential genes within GWAS CAD loci, either by genetic variants or CRISPR perturbations, impairs cellular phenotypes (as measured by FACS) and leads to endothelial dysfunction, a known pathological mechanism for CAD. A recent large-scale trans-ancestry meta-analysis provides additional support to this model. Beside confirming that CAD GWAS loci are enriched for regulatory sequences identified in endothelial cells, the study also found an enrichment of genes with essential functions, such as genes involved in cell cycle progression, division and replication (including *CDKN1A*, the senescence marker used in **Fig 5F**)[5]. Therefore, maybe some of the variants and genes found in our CRISPR screens modestly modulate cellular functions, leading to endothelial dysfunction, atherosclerosis and CAD. Ultimately, the phenotypic characterization of hypomorphic alleles of these essential genes in mouse models (using endothelium-specific targeting techniques) may be needed to address this important question.

We acknowledge several additional limitations of our approach: (1) the six selected cellular readouts may not completely capture how genetic variation associated with CAD influences endothelial functions, (2) Cas9 nuclease can introduce large untargeted truncations, which may disrupt >1 genes [42], (3) Cas9-mediated indels could miss the targeted variants, (4) CRISPRi can silence transcriptional activity over a long distance that can cover many genes, thus complicating the interpretation of the findings [43], and (5) CRISPRa is considered to be mostly effective when targeted to sequences that are proximal to the targeted genes [44]. In particular for CRISPRa, we presented two examples of validated perturbations targeting non-coding variants (*ZNF664*/*CCDC92* and *CNNM2*, **Figs 3B, 6A and 6B**) for which we could not assign causal genes based on transcriptomic analyses. These results cast doubts in using CRISPRa to characterize distal regulatory sequences, especially if it is not supported by orthogonal results. Along the same line, we noted weak correlations of our screen results between CRISPR modalities (**Fig 3A**). While it is possible that certain genomic regions are more amenable to a specific type of perturbation (e.g. coding sequences with Cas9 nuclease), it also possible that some of the CRISPR results seen with a single modality are false positive hits due to the method rather than the screened cellular phenotypes. This needs to be considered carefully when selecting hits for further downstream functional characterization.

Endothelial cell senescence is both a physiological and pathological process [45]. In health, it signals the system for vascular endothelium repair. Senescence also increases with age and in response to traditional CAD risk factors. When it overcomes the regeneration capacity of the system or upon stress, senescence causes endothelial dysfunction and can lead to vascular diseases. Senescent cells accumulate at the sites of atherosclerosis in human blood vessels [46,47] and their selective elimination using transgenic strategies or drugs (senolytics) delays atherogenesis progression in mice [48]. Our data suggest that CAD-associated *DHX38* variants–and potentially variants at other loci awaiting functional characterization–affect key endothelial functions, potentially by inducing premature senescence. This observation links a large body of literature that has implicated senescence in atherosclerosis with variants and genes that modulates endothelial functions. As clinical trials to test the efficacy of senolytics on vascular diseases are now in discussion [49], it will be important to explore whether specific CAD variants or polygenic scores are predictive of their clinical response.

## Materials and methods

### Design of the sgRNA library

We retrieved 92 sentinel genetic variants associated with coronary artery disease (CAD) at genome-wide significant levels (P-value ≤5x10^-8^) from four GWAS meta-analyses available at the time of the design of this experiment [21–24]. For the design of the sgRNA library, we included all sentinel variants as well as variants in strong LD (*r*^2^ >0.8 in the 1000 Genomes Project European-ancestry populations). Because the four large CAD GWAS available when we designed our sgRNA library included mostly individuals from European-ancestry populations, we limited our search for LD proxies to this group. For each variant—sentinel and LD proxy—we identified all possible sgRNA in a 100-bp window centered on the variant itself. Our primary objective in designing this library was to identify high-quality sgRNAs that map as close as possible to the targeted SNPs, independently of genomic annotations. We prioritized sgRNA with the highest predicted quality using the CRISPR OffTarget Tool (version 2.0.3) [50] with a Targeting_guide_score ≥ 20 and the “matches with 0 mismatches” = 1 and “matches with 1 mismatch” = 0 settings. We discarded sgRNA that overlapped heterozygous variants, indels and/or multi-allelic variants in the teloHAEC genome (build hg19). In total, we excluded 895 variants from our screen due to difficulties in designing high quality sgRNAs in their vicinity (86.5% did not pass our sgRNA score threshold, 2.4% overlapped with a heterozygous variant in teloHAEC, and 11.2% both did not pass the quality threshold and overlapped a variant). We selected sgRNA targeting essential genes from a previously published study [51]. For potential positive control genes (*SELE*, *SELP*, *ICAM1*, *VCAM1*, *PECAM1*, *NOS3*, *VWF*, *SOD2*, *SOD3*, *GPX3*, *CAT*, *ITPR1*, *ITPR2*, *ITPR3*, *ATP2A2*, *ATP2A3*, *PLN*, *CAV1*, and *TRPV4*), we selected sgRNA from the Human GeCKOv2 CRISPR knockout pooled library [52]. We also selected sgRNA that targeted the promoter (300-bp window before the transcriptional start site) of positive control genes for the CRISPRa (dCas9-VP64) experiments. For all selected loci (variants, coding sequences, gene promoters), we retained the five top scoring sgRNA for the library design. Finally, we added two sgRNA for the *SELE* locus (SELE_g1, SELE_g2) that we frequently use to validate TNFɑ stimulation. This resulted in a final library of 8051 sgRNA (**S2 Table**).

The sgRNA were synthesized in duplicates by Agilent Technologies (Cat-#: G7555B) to accommodate the specific requirements of the Cas9/dCas9-KRAB and dCas9-VP64 (specific MS2 tracrRNA) experiments. We amplified each specific pool of oligonucleotides as previously described [19], with the following small modifications: we performed two PCR using NebNext High fidelity Master mix (Cat-#: M0541L). The first PCR was used to amplify each pool separately using 2.5ng of pooled oligonucleotides and 500nM of each primer (for the Cas9/dCas9-KRAB library, we used U6_subpool_fwd and Guide_CM_barcode1_rev; for the dCas9-VP64 library, we used U6_subpool_fwd and Guide_MS2_Barcode2_rev). Cycling conditions for PCR1 were 98°C for 30 sec, then 15 cycles of 98°C for 10 sec; 55°C for 10 sec; 72°C for 15 sec and a final step of 72°C for 2 min and a 10°C hold. We performed the second PCR to add homologous sequences, using the U6_screen_fwd and Tracr_rev oligonucleotides for the Cas9/dCas9-KRAB library, and the U6_screen_fwd and Tracr_MS2_rev oligonucleotides for the dCas9-VP64 library, in both cases using ⅕ of PCR1 as template. Cycling conditions for PCR2 were 98°C for 30 sec, then 10 cycles of 98°C for 10 sec; 55°C for 10 sec; 72°C for 15 sec and a final cycle of 72°C for 2 min and 10°C hold. See table **S9 Table** for primer details.

After gel extraction and PCR purification, we performed Gibson assembly in both respective vectors (pHKO9-Neo and lentisgRNA(MS2)-zeo backbone addgene 61427). For pHKO9-Neo, we replaced the Crimson fluorescent gene in the pHKO9-Crimson-CM vector (gift from Dan Bauer’s lab) by a neomycin resistance (NeoR) sequence. Briefly, we amplified the NeoR gene from our pCas9-Neo vector [53] using BsiWI-Neo-Fwd and MluI-Neo_rev primer (**S9 Table**). After digestion by BsiWI and MluI, we cloned the segment in pHKO9-Crimson_CM, which had been digested with BsiWI and MluI. We amplified each library using ten independent maxi-preparations (Macherey-Nagel cat# 740424). To control the quality of both libraries, we sequenced them on an Illumina HiSeq4000 instrument and calculated the Gini index, which summarizes read distribution across sgRNA in a given pool. For a good-quality sgRNA library, the expected Gini index is ≤0.2, and we obtained Gini indexes of 0.050 and 0.052 for the Cas9/dCas9-KRAB and dCas9-VP64 library, respectively.

### Engineering of teloHAEC cell lines to stably express Cas9 variants or base editors

TeloHAEC are immortalized human aortic endothelial cells obtained by over-expressing telomerase (ATCC CRL-4052). These cells have a normal female karyotype (46;XX) and exhibit many of the properties and functions of human vascular endothelial cells [7]. We previously showed that the teloHAEC transcriptional and epigenomic responses to TNFα treatment is highly correlated with the responses of primary human coronary artery endothelial cells to the same stimulation [7]. We cultivated teloHAEC in Vascular Cell Basal Medium supplemented with Vascular Endothelial Cell Growth kit-VEGF (ATCC through Cedarlane PSC-100-030 and PSC-100-041). We generated our teloHAEC cells models expressing either Cas9, dCas9-KRAB or dCas9-VP64 + MPHv2 using Addgene vectors #52962, #46911, #61425 and #89308, and base editors using plenti-U6-gRNAentry-EFS-ABE8e-(D10A)SpRY-P2A-Blast, called telo-HAEC ABE8e-SpRY. We carried out lentiviral infection as previously described [53].

### Pooled CRISPR screen experiments

We produced four batches of lentiviruses for each sgRNA library pool (Cas9/dCas9-KRAB, dCas9-VP64). We infected each teloHAEC cell line (Cas9, dCas9-KRAB, dCas9-VP64) at a multiplicity of infection of 0.3 using each batch of viruses separately. Following viral infection, we selected cells using zeocin (teloHAEC-dCas9-VP64) or G418 (teloHAEC-Cas9/-dCas9-KRAB) for five (teloHAEC-dCas9-VP64) or seven days (teloHAEC-Cas9/-dCas9-KRAB) in vascular cell basal medium (ATCC PCS-100-030) to remove any cells that did not incorporate a vector. After selection, we stimulated cells expressing Cas9 or dCas9-KRAB using TNFɑ (10ng/μl) for four hours to induce a pro-inflammatory response; we did not stimulate cells expressing dCas9-VP64, reasoning that the VP64 transcriptional domain should activate gene expression. Following TNFɑ stimulation, we immunostained cells (around 50M cells) with antibodies linked to phycoerythrin for adhesion molecules (E-selectin (BD BIOSCIENCES Cat-#: 551145), VCAM-1 (Cat-#: 12-1069-42), ICAM-1 (Cat-#: 12-0549-42)) or we incubated with fluorescent dye-based reagents for endothelial signaling markers: (nitric oxide (NO) (DAF-FM Diacetate, Cat-#: D23844)), reactive oxygen species (ROS) (CM-H2DCFDA, Cat-#: C6827), calcium signaling (Fura Red, Cat-#: F3021)). We calibrated the FACS assays with positive control treatments to make sure that we could robustly detect changes in the measured phenotypes. Antibodies and fluorescent dye-based reagents were titrated to use optimal concentrations. We also quantified how teloHAEC were responding to ionomycin for calcium signaling, to sodium nitroprusside for NO and to TNFα for reactive oxygen species. For adhesion molecules, we utilized sgRNA targeting coding exons and promoter regions of *SELE*, *ICAM1* and *VCAM1* as positive controls. Unless otherwise stated, we purchased all antibodies and dyes from ThermoFisher Scientific. Subsequently, we sorted stained cells by flow cytometry on a BD FACSARIA FUSION flow cytometer to collect the top and bottom 10% of fluorescently labeled cells. For each experiment, we infected 20-50M cells at a multiplicity of infection (MOI) of 0.3 (740–1860 cells/sgRNA) and analyzed a similar number of cells (20-50M) for FACS, resulting in approximately 500 to 1200 cells/sgRNA. FACS traces were generated with FlowJo (BD Biosciences). We extracted genomic DNA from both top and bottom 10% cell fractions separately (around 5M cells in each fraction) using the QIAGEN DNeasy Blood and Tissue kit (Cat No. 69504) according to manufacturer’s instructions.

### Amplification and sequencing of pooled CRISPR experiments

We amplified sgRNA sequences from genomic DNA via PCR, followed by a cleanup step using the QIAGEN QIAquick PCR purification kit (Cat-#: 28104) according to the manufacturer’s instructions. We used the primer sequences and PCR settings as previously described in ref. [19]. We created sequencing libraries using Illumina TruSeq adapters according to the manufacturer’s protocols. We sequenced the libraries on an Illumina Hiseq4000 instrument at the McGill University and Genome Quebec Innovation Centre (MUGQIC). Generally 6 samples were multiplexed per sequencing lane for a target read coverage of ~500 reads per sgRNA per sample (**S5B Fig**).

### Computational analysis of pooled CRISPR screen data

We processed raw sequencing data from the BCL to the FASTQ format using bcl2fastq at MUGQIC. Raw FASTQ reads were quality-controlled using FastQC (https://www.bioinformatics.babraham.ac.uk/projects/fastqc/) and MultiQC [54]. We performed downstream analysis of sgRNA sequencing data using MAGECK (v.0.5.9) [55]. We quantified sgRNA sequences using MAGECK count against the list of sgRNA sequences in the library (**S2 Table**), allowing for no mismatches in the sgRNA sequence. We then tested the difference in sgRNA counts between the bottom and top 10% flow cytometry fractions for each readout using MAGECK maximum likelihood estimation (*mle*) method with median normalization [56]. Predicted functional impact of variants and overlap with genomic features was computed using VEP [57], biomart and Goldmine in R [58–60].

### UMAP representation of sample-level count data

We normalized raw sgRNA counts using variance stabilizing transformation (vst) in DESeq2 [61]. To account for baseline differences between plasmid preparations, we further normalized samples to their respective vector library by dividing the vst normalized sgRNA count by the vst normalized count of the Cas9/dCas9-KRAB or dCas9-VP64 library, respectively. We calculated principal components using the top 10% most variable sgRNA (805 sgRNA) across all cell sorted samples based on normalized counts. Next, we used the loadings from the first three principal components in Uniform Manifold Approximation and Projection (UMAP) [62] to create a two-dimensional embedding of the normalized sgRNA count data. Each dot in the UMAP plot represents one sequenced sample (top or bottom 10% of stained cells).

### Analysis of sgRNAs targeting essential genes

To test for potential effects of sgRNA on endothelial cell death and proliferation, we compared sgRNA counts of all samples across the same cellular model (Cas9, dCas9-KRAB, dCas9-VP64) against the respective baseline vector library sgRNA count using MAGECK *mle*. We used sgRNAs targeting essential genes in the teloHAEC Cas9 cellular model as positive controls.

### Single sgRNA validation

We individually cloned each sgRNA for validation as previously described [63]. We produced lentiviruses, infected cells, performed antibiotic selection, and stained cells as for the pooled CRISPR screen. We analyzed cells using flow cytometry (BD FACSCelesta (BD Biosciences, San Jose, CA, USA) equipped with a 20 mW blue laser (488 nm), a 40 mW red laser (640 nm), and a 50 mW violet laser (405 nm). For each experiment, we measured the mean fluorescent intensity (MFI) obtained for sgRNA of interest and compared it with the MFI for control sgRNA (safe-harbor and/or scrambled sgRNA). Safe-harbor sgRNA sequences were based on Pellenz et al. [64]. We performed each experiment at least three times. For statistical analyses, we used Student’s t-test and determined that a sgRNA had a significant effect on the measured phenotype when a one-tailed P-value ≤0.05.

For *DHX38* Crimson experiments, we individually cloned each sgRNA (2 different guides were used for DHX38 (sg10966 and sg11664) and 2 for Safe-Harbor, respectively) in pHKO9-Crimson-CM vector (gift from Dan Bauer’s lab). We produced lentiviruses, infected cells and performed flow cytometry on a BD FACSARIA FUSION flow cytometer at day 2, day 4 and day 7 post-infection. We analyzed the percentage of Crimson positive cells and we sorted Crimson positive and negative cells to extract RNA in each fraction. We extracted total RNA using RNeasy Plus Mini Kit (Qiagen cat #: 74136). We measured RNA integrity and concentration using Agilent RNA 6000 Nano II assays (Agilent Technologies) on an Agilent 2100 Bioanalyzer and Take3 on Cytation V (Biotek). We reverse transcribed 750ng of total RNA using random primers and 1 U of the MultiScribe Reverse Transcriptase (Applied Biosystems) in a 20 μL reaction volume at 100 mM dNTPS and 20 U of RNase inhibitor with these three steps: 10 min at 25°C, 120 min at 37°C and 5 min at 85°C. We followed the MIQE guidelines to assess quality and reproducibility of our qPCR results [65]. We performed qPCR in triplicates for all samples using: 1.25 μL of cDNA (1/50 dilution), 5 μL of Platinum SYBR Green qPCR SuperMix-UDG (Life Technologies) and 3.75 μL of primer pair mix at 0.8 μM on a CFX384 from Biorad. We used the following thermal profile: 10 min at 95°C, and 40 cycles of 30 s at 95°C, 30 s at 55°C and 45 s at 72°C. We carried out melting curve analyses after the amplification process to ensure the specificity of the amplified products. We also simultaneously performed qPCR reactions with no template controls for each gene to test the absence of non-specific products. Cq values were determined with the CFX Manager 3.1 (Bio-Rad) software and expression levels were normalized on the expression levels of the house-keeping genes TATA-box binding protein (*TBP*), hypoxanthine-guanine phosphoribosyltransferase (*HPRT*), and glyceraldehyde 3-phosphate dehydrogenase (*GAPDH*) using the ΔΔCt method. The primer sequences are in **S9 Table**.

### Single-guide RNP validation in human primary endothelial cells

We nucleofected Human Coronary Artery Endothelial Cells (HCAEC) (Lonza through Cedarlane CC-2585) using Nucleofector 4D and P5 Primary cell 4D Nucleofector X kit S (Lonza, V4XP-5032) according to the supplier’s recommendations. First, we warmed-up 2 ml of media without antibiotics (same as for teloHAEC) per condition in a 6-wells plate format. We annealed crRNA and TracrRNA (from IDT, Alt-R CRISPR-Cas9 crRNA XT and Alt-R CRISPR-Cas9 tracrRNA) in nuclease duplex buffer at 30uM, 5 min at 95°C then cool down to room temperature to obtain gRNA. We incubated 6ul of the gRNA (2 different guides were used for DHX38 (sg10966 and sg11664) and 2 for Safe-Harbor, respectively) with 1ul of Cas9 (IDT, Alt-R S.p. Cas9 Nuclease V3) diluted ⅓ and 18ul of P5 solution for 10 minutes at RT. We added 300K cells resuspended in 5ul of supplemented P5 buffer and mixed with the 25ul of RNP complex. We used protocol EH-100 for nucleofection. Afterward, we added 70ul of media without antibiotics per condition and transferred 100ul of each condition per well. We changed the media 24 hrs later and passed them on day 4 to extract gDNA/RNA and cultivated them for another 3 days. We also extracted gDNA/RNA on day 7. gDNA and RNA/cDNA and qPCR are performed as described above.

### PCR for determination of CRISPR-Cas9-induced indels or base editing efficiency

We isolated gDNA using QuickExtract DNA Extraction Solution (Epicentre, QE0905) from 1x10^5^ cells. We used 100 or 200 ng of gDNA as a template for PCR reaction with the corresponding primers (see **S9 Table**). gDNA from parental teloHAEC cells was used as control. Obtained PCR products were analysed by electrophoresis on a 1% agarose gel prior to Sanger sequencing. We used TIDE (tracking of indels deconvolution) software for analysis [66].

### Base editing of rs12906125

We infected our teloHAEC ABE8e-SpRY population with a guide in rs12906125 locus, CGGGACGGTCGGGCCGGTCC, cloned in pHKO9-Neo-CM as previously described [63]. teloHAEC are endogenously heterozygous at rs12906125 (A/G) and the treatment with ABE8e-SpRY should edit the genotype to G/G. Because of multiple Cs near the SNP, it was not possible to engineer clear edits towards the A/A genotype using cytosine base editors. After 3 weeks of proliferation, we derived clones by limiting dilution and extracted gDNA for PCR. We performed PCR as already described in the previous section with primers sequences (see **S9 Table)** and we used EditR software for analysis [67]. 45 clones out of 60 were analyzed by EditR and 15 out of 45 were edited (perfect edit, with the A/G genotype at rs12906125 edited to the G/G genotype, ~30% editing efficiency). We extracted also RNA to perform *FES* and *FURIN* mRNA expression by qPCR. RNA extraction, cDNA and qPCR we generated as described in the previous section. We compared expression with and without TNFα (10ng/μl) treatment for four hours.

### Assays cell senescence

Using the same experimental design (DHX38-Crimson), we performed beta-galactosidase staining using the CellEvent Senescence Green Flow Cytometry Assay Kit from Invitrogen on day 2, day 4 and day 7 following the manufacturer’s protocol. Briefly, we trypsinized and we fixed the cells with 2% paraformaldehyde solution for 10 minutes at room temperature, washed them in 1%BSA/PBS and incubated for 1h30 in 1/500 working solution. After incubation, we washed the cells with 1%BSA/PBS and analyzed them by flow cytometry. We measured the β-galactosidase fluorescence signal in positive and negative Crimson cells independently. As positive control, non-infected cells were treated with 20 μM of Etoposide (Sigma, E1383-25) for 2, 4 and 7 days.

### Transcriptome data analysis

For RNA-seq analysis, we extracted RNA using RNeasy plus mini kit from Qiagen (cat #: 74136). RNA-seq experiments were carried out by the Centre d’Expertise et de Services Genome Quebec using rRNA-depleted TruSeq stranded (HMR) libraries (Illumina) on an Illumina Hiseq 4000 instrument (paired-ends, 100-bp reads) and by The Center for applied Genomics (Toronto) using rRNA-depletion library prep on an Illumina NovaSeq-SP flow cell. We quality-controlled raw fastq files with FastQC and multiQC [67]. We used kallisto (v. 0.46.0) to quantify transcript abundances [68] against ENSEMBL reference transcripts (release 94) followed by tximport to calculate gene-level counts [69]. We utilized regularized log-transformation (rlog) in DESeq2 [61] as input for principal component analysis (PCA). DESeq2 [69] was further used to identify differentially-expressed genes between teloHAEC cell models (Cas9, dCas9-VP64) infected by lentiviruses with safe-harbor sgRNA or sgRNA identified in the pooled CRISPR screens. We excluded genes expressed with less than 10 reads across all samples from the analysis. We performed shrinkage for effect size estimates using apeglm using the lfcShrink method [70]. Genes differentially expressed with a Benjamini-Hochberg adjusted p-value ≤ 0.05 were considered significant (**S10 Table**). Gene set enrichment analysis was performed using the R package fgsea using 100,000 permutations against the Hallmark gene sets from msigdbr (https://igordot.github.io/msigdbr/) [71,72]. We quantified short indels in the RNA-seq data of *DHX38* (sgRNA_10966) and *MAT2A* (sgRNA_02249) using the tools transIndel and Genesis-Indel, which are specifically designed to identify indels in the unmapped read fraction of samples [73,74].

### Overexpression of open reading frames (ORF) in teloHAEC

We obtained pEntry vectors containing the *CCDC92* ORF from John D. Rioux’s lab. First, we cloned the gateway cassette from pLVX-EF1α-attR1-ccdB-attR2-IRES-puro-emGFP digested with XbaI (NEB, cat no R0145S) and ligated with Quick ligase from NEB (cat no M2200) in pLVX-EF1α-IRES-mCherry (refer as Empty-mCherry thereafter) also digested by XbaI and dephosphorylated using Fast AP (ThermoFisher cat no FEREF0654). Briefly, we used 50ng of digested vector and a ratio of 3:1 of insert. We performed ligation for 15 min at room temperature and then we transformed 2μL of ligation in One shot ccdB Survival 2 T1 Competent cells (Life Technologies, cat no A10460) and plated on LB agar ampicillin plates. Both vectors were gifts from John D. Rioux’s lab. We individually cloned each ORF using Gateway LR Clonase II Enzyme mix protocol (Life Technologies, cat no 11791020) in the new pLVX-EF1α-attR1-ccdB-attR2-IRES-mCherry vector. Briefly, we used 300ng of each pEntry/pDONR ORF vector with 300 ng of pLVX-EF1α-attR1-ccdB-attR2-IRES-mCherry with 4μL of 5X LR buffer and TE pH8.0 to 16 μL, then we added 4 μL of LR enzyme and incubated for 1 hr at 25°C. After this incubation, we added 2 μL of proteinase K and incubated for another 10 min at 37°C. We transformed 1 μL of each reaction in One Shot Stbl3 Chemically Competent *E*. *coli* (Life Technologies, cat no C7373-03) and plated on LB agar ampicillin plates. All vectors and ORF sequences have been validated by Sanger sequencing.

### Analysis of scRNA-seq data from human coronary arteries

Single-cell gene expression matrix from human right atherosclerotic coronary arteries (three male and one female donors), was downloaded from NCBI GEO (GSE131780, https://www.ncbi.nlm.nih.gov/geo/query/acc.cgi?acc=GSE131780). The data was re-analyzed using the Seurat package in R with a standard single-cell clustering pipeline. Gene expression data was normalized using the SCTransform function from Seurat (v.3.2.3), regressing out the percentage of mitochondrial gene expression. Principal components analysis was performed, followed by dimensional reduction with Uniform Manifold Approximation and Projection (UMAP) using the first 20 principal components as input. Gene expression was visualized on the first two UMAP dimensions using the kernel density function (plot_density) from the Nebulosa package (v.0.99.92)[75] for endothelial cell marker and candidate genes.

### Statistics and data analysis

Unless noted otherwise, we performed all data and statistical analyses in R (v.3.6.0) using Rstudio. We ran our analyses on a high performance computing cluster (Beluga) from Calcul Quebec/Compute Canada. For MAGECK variant-level analyses, permutation-based FDR of ≤10% were considered significant. For RNA-seq analysis, genes with a Benjamini-Hochberg adjusted P-value in DESeq2 ≤0.05 were considered significant [75]. For the co-localization analyses at the *DHX38* locus, we used the coloc package [76] and publicly available summary statistics from large LDL-C [77] and CAD GWAS [77].

## Supporting information

S1 Fig Fractions of alleles (y-axis) mediated by CRISPR/Cas9 that disrupt the coronary artery disease (CAD)-associated targeted variants in the pooled CRISPR screens for endothelial functions. On the x-axis, we report the distance between the CAD variants and the PAM sites. We used the FORECAST algorithm to perform this analysis (Allen et *al*., Nature Biotech., 2019).
(PNG)

S2 Fig Flow cytometry profiles for all six endothelial cell readouts with Cas9. For each experiment, we show a representative figure of the flow cytometry profiles obtained for each of the four lentiviral batches (1a-1d) used in our experiments. Red: Non-infected, non-stained cells; Blue: Non-infected, stained cells; Orange: Infected and stained cells. For E-selectin, ICAM1 and VCAM1, the fluorochrome is PE; for ROS, NO and Ca^2+^, the fluorochrome is FITC.
(PDF)

S3 Fig Flow cytometry profiles for all six endothelial cell readouts with CRISPRi. For each experiment, we show a representative figure of the flow cytometry profiles obtained for each of the four lentiviral batches (1a-1d) used in our experiments. Red: Non-infected, non-stained cells; Blue: Non-infected, stained cells; Orange: Infected and stained cells. For E-selectin, ICAM1 and VCAM1, the fluorochrome is PE; for ROS, NO and Ca^2+^, the fluorochrome is FITC.
(PDF)

S4 Fig Flow cytometry profiles for all six endothelial cell readouts with CRISPRa. For each experiment, we show a representative figure of the flow cytometry profiles obtained for each of the four lentiviral batches (1a-1d) used in our experiments. Red: Non-infected, non-stained cells; Blue: Non-infected, stained cells; Orange: Infected and stained cells. For E-selectin, ICAM1 and VCAM1, the fluorochrome is PE; for ROS, NO and Ca^2+^, the fluorochrome is FITC
(PDF)

S5 Fig Quality-control metrics of sequenced lentiviral libraries. (**A**) The Gini index is a measure of sgRNA diversity across a sample. A low Gini index indicates that all sgRNAs are equally represented in the sequenced data. The data is stratified (color-coded) by Cas9 proteins. For the sequence data across all experiments, the Gini index is below the recommended threshold (Gini index <0.2,^65^. The *y*-axis denotes the number of sgRNAs with 0 reads for each experiment. (**B**) Density distribution of reads per sgRNA for all endothelial phenotypes and Cas9 proteins (the x-axis is read depth on a log_10_ scale).
(PDF)

S6 Fig Graphical representation of three genes that encode adhesion molecules important for monocyte rolling and attachment at the onset of atherosclerosis. Locus views for adhesion molecule positive control genes (**A**) *SELE* (E-selectin), (**B**) *VCAM1* and (**C**) *ICAM1*. We also represent the position of the sgRNAs that were tested in the pooled CRISPR screens (GECKO, coding sequences; SAM and promoter, regulatory sequences), as well as RNA-seq and ATAC-seq data in teloHAEC that are un-stimulated or activated for 4 hours with TNFɑ (Lalonde et *al*., Genome Biol., 2019).
(PDF)

S7 Fig *SELE*/E-selectin expression in teloHAEC with genotypes A/G or G/G at rs12906125 in the *FES* promoter. For all experiments, cells were treated for 4 hours with TNFα. (A) *SELE* mRNA expression levels measured by real-time qPCR. We analyzed at least six clones of each genotype. Based on the CRISPRa (**Fig 3B**) and *FES* qPCR experiments (**Fig 4C**), we expect G/G cells to express *SELE* at higher levels. The difference is not significant (Student’s *t*-test *P* = 0.17). (**B**) We used flow cytometry to measure E-selectin at the cell membrane of three teloHAEC clones with the A/G genotype and three with the G/G genotype. The difference in mean fluorescence intensity (MFI) between the two groups is non-significant (one-tailed Student’s *t*-test *P* = 0.42).
(PNG)

S8 Fig Flow cytometry experiments for E-selectin using nucleofection of ribonucleoprotein (RNP) complexes containing Cas9 and sg_10966 (rs2074626 in *DHX38*). Results are shown for two independent biological replicates. At the 10% bottom fraction threshold for E-selectin levels in the control experiments (empty nucleofection, black), we find 24% (panel A) and 31% (panel B) of cells following Cas9 RNP targeting rs2074626 in *DHX38*. This result confirms that *DHX38* inactivation reduces E-selectin presentation at the cell membrane. The fluorochrome is PE.
(PDF)

S9 Fig Validation of the effect of *DHX38*-targeting sgRNAs on the expression levels of *DHX38* and *CDKN1A* in primary human coronary artery endothelial cells (HCAEC) cells using ribonucleoprotein (RNP) Cas9 complexes. Nucleofection of HCAEC with sgRNAs targeting *DHX38* coding exons decrease *DHX38* and increase *CDKN1A* expression levels (4 and 7 days post nucleofection). The sgRNA against *LPL* was not significant in our pooled CRISPR screen and is used here as a negative control, along with the safe harbor sgRNAs. N.S. = not significant.
(PNG)

S10 Fig CRISPRa experiment with a silent sgRNA at the *LPL* locus does not influence cell proliferation nor the expression of senescence marker genes. (**A**) teloHAEC that express dCas9-VP64 were infected with a lentivirus that carries a sgRNA that targets rs1441755 at the LPL locus. This sgRNA was silent in all our pooled CRISPR screens for all six endothelial phenotypes tested. In the absence or presence of antibiotic selection (Zeocin), LPL_sg08237 does not affect cell proliferation. Cell counts are mean ± standard deviation of 10 and 5 images for the safe harbor and LPL_sg08237 sgRNAs, respectively. The differences are not significant (Student’s *t*-test *P*>0.06). (**B**) Expression of *CCDC92* and *CDKN1A* in teloHAEC that express dCas9-VP64 (with zeocin selection). Results are mean ± standard deviation from 4 and 2 experiments for the safe harbor and LPL_sg08237 sgRNAs, respectively. The differences are not significant (Student’s *t*-test *P*>0.6).
(PDF)

S11 Fig Over-expression of the *CCDC92* open reading frame (ORF) in sorted teloHAEC cells. Ectopic over-expression of the *CCDC92* ORF via viral infection alongside non-infected (NI) and empty controls (Empty) (top *x*-axis). For each over-expression experiment, we sorted cells by flow cytometry between mCherry negative (neg) and mCherry positive (pos) cells, and quantify transcripts by quantitative PCR in each fraction individually. Over-expression of the *CCDC92* ORF causes a strong induction of CCDC92 expression (pos, top row, blue bars). However, the increase expression in ICAM1 levels is not significant when comparing mCherry positive (*i*.*e*. over-expressing *CCDC92*) and negative cells (bottow row, blue bars).
(PDF)

S1 Table List of sentinel variants associated with coronary artery disease (CAD) and their proxy variants in strong linkage disequlibrium (LD) based on genotypes from 1000 Genomes Project populations of European ancestry. Chromosomal positions are on build hg19 of the human genome. REF and ALT correspond to reference and alternate alleles. Rsq is the measure of LD. Distance is the distance between the sentinel and the proxy variants (in base pairs).
(XLSX)

S2 Table We used the CRISPR Off-Target Tool (http://www.mhi-humangenetics.org/en/resources/) to score candidate sgRNAs. sgRNA group: Reason of sgRNA inclusion. SNP/Gene: Targeted locus. sgRNA_ID: The sgRNA ID. sgRNA_Seq: The sgRNA sequence. Targeting_guide_score: This score assumes that the sgRNA should have one genomic match that is the intended target. If there is no perfect match, this score will be equal to the non-targeting score. Non_targeting_score: This score assumes that the sgRNA should NOT match any sequence of the genome. best_match_score: Score of best match. best_match_position: Position of best match. best_match_sequence: Sequence of best match. best_match_CFD: CFD (cutting frequency determination) score of best match. mean_CFD: Mean CFD score. median_CFD: Median CFD score. max_CFD: Maximum CFD score. min_CFD: Minimum CFD score. perc_CFD: 10, 25, 75 and 90th percentile of CFD score. Total_matches: Total number of matches. N_X: These columns contain the number of matches with X mismatches. There will be X+1 columns ranging from N_0 (perfect match) to N_X, where X is the maximum number of allowed mismatches. Genomic positions are on build hg19.
(XLSX)

S3 Table For each GWAS coronary artery disease (CAD) locus, we report the number of variants in linkage disequilibrium (LD) for which we designed (Variants designed) and experimentally tested (Variants tested) at least 2 sgRNAs. The Variant saturation number is caculated by dividing Variants tested by Variants designed. "1" in the column Locus with 1 hit indicated that at least one of the variants tested was significant in the CRISPR screens. For each GWAS locus, we list the total number and identifiers of the hits in the CRISPR screens. We report the closest genes and the assigned biological pathways based on Klarin et al, Nature Genetics, 2017.
(XLSX)

S4 Table Variant-level results for all pooled CRISPR screens for endothelial cell readouts. nr_sgRNA, number of sgRNAs that targeted the variant/gene; Beta, SE, zscore, pvalue and false discovery rate (FDR) are statistics generated by MAGECK; FACS_phenotype, cellular readouts. The Beta corresponds to the effect when comparing sgRNA normalized counts between the top and the bottom 10% FACS fractions. For each variant, we provide the Variant Effect Predictor’s most severe annotation. We also annotated variant if they map (1 = overlap) to ATAC-seq peaks in resting teloHAEC (NT) or teloHAEC stimulated for 4h or 24h with TNFalpha. The ATAC-seq data is from Lalonde et al., Genome Biology, 2019. We also used chromatin states predictions from endothelial cells from the EpiMap Project to annotate all tested variants (Boix et al., Nature, 2021). The definition of each EpiMap cell type and chromatin state can be found at: https://personal.broadinstitute.org/cboix/epimap/metadata/Short_Metadata.html and https://www.nature.com/articles/s41586-020-03145-z/figures/7. We used the following chromatin states to define enhancers: EnhG1, EnhG2, EnhA1, EnhA2, EnhWk. Although we present results for all SNPs tested in our screens, our donwstream analyses only considered SNPs with FDR<10% that were tested with at least 2 high-quality sgRNAs.
(XLSX)

S5 Table Correlation between sgRNA-level count between replicates for each Cas9 modality and FACS cellular readout. (XLSX)

S6 Table Validation results for sgRNAs that were highlighted in the pooled CRISPR screens and selected for further characterization. For each SNP, we characterized the sgRNA with the best overall performance in the CRISPR screens. For each sgRNA, we combined results from 3–4 biological replicates. Within each replicate, we compared the overall distributions of the endothelial cell readouts (between SNP sgRNA and negative control sgRNA) using the Kolmogorov-Smirnov test (Fig 3B). To combine results across replicates and assess significance, we used a simple one-tailed Student’s t-test. MFI, mean fluorescence intensity from the flow cytometer.
(XLSX)

S7 Table Gene-set enrichment analysis results from fgsea (https://www.bioconductor.org/packages/release/bioc/html/fgsea.html) for all RNA-seq experiments against the Hallmark gene sets from MigSigDB after 100000 permutations per experiment. Enrichment results represent enrichment of differentially expressed genes of candidate sgRNA against safe harbor controls for the specificed Cas9 variant (Cas9, CRISPRa). ES, enrichment score; NES, enrichment score normalized to mean enrichment of random samples of the same size; nMoreExtreme, a number of times a random gene set had a more extreme enrichment score value; size of the pathway after removing genes not present in the dataset; leadingEdge, vector with indexes of leading edge genes that drive the enrichment.
(XLSX)

S8 Table Quantification by Tracking of Indel by Decomposition (TIDE) of insertion-deletions (indels) due to DHX38 sg10966 or sg11664 in teloHAEC stably over-expressing Cas9. Indels were only detected in CRIMSON+ cells. We never observed DHX38 indels in teloHAEC infected with safe harbor sgRNA. Data from three replicates at days 2, 4 and 7 post-infection are summarized. TIDE captures the size of the indels as well as their frequency in the cell population. NA, not available. None, no indels were detected.
(XLSX)

S9 Table Oligonucleotide sequences used throughout this project. (XLSX)

S10 Table Results for DESeq2 differential expression analysis for 5 validation RNA-seq experiments of candidate sgRNA against safe-harbor controls. baseMean: mean of normalized counts across all samples, log2FoldChange: log2 fold-change of candidate sgRNA over safe-harbor sgRNA read counts, lfcSE: standard error estimate of log2 fold-change, pvalue: nominal p-value, padj: Benjamini-Hochberg adjusted p-value, gene: human gene symbol (Ensemble 94), experiment: RNA-seq validation experiment identifier. Differential expression results were calculated for each experiment separately.
(TSV)

## References

[1] Hopkins PN. Molecular biology of atherosclerosis. Physiol Rev. 2013;93:1317–542. doi: 10.1152/physrev.00004.2012 23899566

[2] Libby P, Ridker PM, Hansson GK. Progress and challenges in translating the biology of atherosclerosis. Nature. 2011;473:317–25. doi: 10.1038/nature10146 21593864

[3] van der Harst P, Verweij N. Identification of 64 Novel Genetic Loci Provides an Expanded View on the Genetic Architecture of Coronary Artery Disease. Circ Res. 2018;122:433–43. doi: 10.1161/CIRCRESAHA.117.312086 29212778 PMC5805277

[4] Musunuru K, Kathiresan S. Genetics of Common, Complex Coronary Artery Disease. Cell. 2019;177:132–45. doi: 10.1016/j.cell.2019.02.015 30901535

[5] Tcheandjieu C, Zhu X, Hilliard AT, Clarke SL, Napolioni V, Ma S, et al. Large-scale genome-wide association study of coronary artery disease in genetically diverse populations. Nat Med. Springer Science and Business Media LLC; 2022;28:1679–92. doi: 10.1038/s41591-022-01891-3 35915156 PMC9419655

[6] Krause MD, Huang R-T, Wu D, Shentu T-P, Harrison DL, Whalen MB, et al. Genetic variant at coronary artery disease and ischemic stroke locus 1p32.2 regulates endothelial responses to hemodynamics. Proc Natl Acad Sci U S A. 2018;115:E11349–58. doi: 10.1073/pnas.1810568115 30429326 PMC6275533

[7] Lalonde S, Codina-Fauteux V-A, de Bellefon SM, Leblanc F, Beaudoin M, Simon M-M, et al. Integrative analysis of vascular endothelial cell genomic features identifies AIDA as a coronary artery disease candidate gene. Genome Biol. 2019;20:133. doi: 10.1186/s13059-019-1749-5 31287004 PMC6613242

[8] Cahill PA, Redmond EM. Vascular endothelium—Gatekeeper of vessel health. Atherosclerosis. 2016;248:97–109. doi: 10.1016/j.atherosclerosis.2016.03.007 26994427 PMC6478391

[9] Godo S, Shimokawa H. Endothelial Functions. Arterioscler Thromb Vasc Biol. 2017;37:e108–14. doi: 10.1161/ATVBAHA.117.309813 28835487

[10] Sandoval R, Malik AB, Minshall RD, Kouklis P, Ellis CA, Tiruppathi C. Ca(2+) signalling and PKCalpha activate increased endothelial permeability by disassembly of VE-cadherin junctions. J Physiol. 2001;533:433–45. doi: 10.1111/j.1469-7793.2001.0433a.x 11389203 PMC2278647

[11] Gorgoulis V, Adams PD, Alimonti A, Bennett DC, Bischof O, Bishop C, et al. Cellular Senescence: Defining a Path Forward. Cell. 2019;179:813–27. doi: 10.1016/j.cell.2019.10.005 31675495

[12] Wiley CD, Flynn JM, Morrissey C, Lebofsky R, Shuga J, Dong X, et al. Analysis of individual cells identifies cell-to-cell variability following induction of cellular senescence. Aging Cell. 2017;16:1043–50. doi: 10.1111/acel.12632 28699239 PMC5595671

[13] Hernandez-Segura A, de Jong TV, Melov S, Guryev V, Campisi J, Demaria M. Unmasking Transcriptional Heterogeneity in Senescent Cells. Curr Biol. 2017;27:2652–2660.e4. doi: 10.1016/j.cub.2017.07.033 28844647 PMC5788810

[14] Teo YV, Rattanavirotkul N, Olova N, Salzano A, Quintanilla A, Tarrats N, et al. Notch Signaling Mediates Secondary Senescence. Cell Rep. 2019;27:997–1007.e5. doi: 10.1016/j.celrep.2019.03.104 31018144 PMC6486482

[15] Acosta JC, Banito A, Wuestefeld T, Georgilis A, Janich P, Morton JP, et al. A complex secretory program orchestrated by the inflammasome controls paracrine senescence. Nat Cell Biol. 2013;15:978–90. doi: 10.1038/ncb2784 23770676 PMC3732483

[16] Gimbrone MA Jr, García-Cardeña G. Endothelial Cell Dysfunction and the Pathobiology of Atherosclerosis. Circ Res. 2016;118:620–36. doi: 10.1161/CIRCRESAHA.115.306301 26892962 PMC4762052

[17] Wright AV, Nuñez JK, Doudna JA. Biology and Applications of CRISPR Systems: Harnessing Nature’s Toolbox for Genome Engineering. Cell. 2016;164:29–44. doi: 10.1016/j.cell.2015.12.035 26771484

[18] Doench JG, Hartenian E, Graham DB, Tothova Z, Hegde M, Smith I, et al. Rational design of highly active sgRNAs for CRISPR-Cas9-mediated gene inactivation. Nat Biotechnol. 2014;32:1262–7. doi: 10.1038/nbt.3026 25184501 PMC4262738

[19] Joung J, Konermann S, Gootenberg JS, Abudayyeh OO, Platt RJ, Brigham MD, et al. Genome-scale CRISPR-Cas9 knockout and transcriptional activation screening. Nat Protoc. 2017;12:828–63. doi: 10.1038/nprot.2017.016 28333914 PMC5526071

[20] Fulco CP, Munschauer M, Anyoha R, Munson G, Grossman SR, Perez EM, et al. Systematic mapping of functional enhancer-promoter connections with CRISPR interference. Science. 2016;354:769–73. doi: 10.1126/science.aag2445 27708057 PMC5438575

[21] Nikpay M, Goel A, Won H-H, Hall LM, Willenborg C, Kanoni S, et al. A comprehensive 1,000 Genomes-based genome-wide association meta-analysis of coronary artery disease. Nat Genet. 2015;47:1121–30. doi: 10.1038/ng.3396 26343387 PMC4589895

[22] Howson JMM, Zhao W, Barnes DR, Ho W-K, Young R, Paul DS, et al. Fifteen new risk loci for coronary artery disease highlight arterial-wall-specific mechanisms. Nat Genet. 2017;49:1113–9. doi: 10.1038/ng.3874 28530674 PMC5555387

[23] Klarin D, Zhu QM, Emdin CA, Chaffin M, Horner S, McMillan BJ, et al. Genetic analysis in UK Biobank links insulin resistance and transendothelial migration pathways to coronary artery disease. Nat Genet. 2017;49:1392–7. doi: 10.1038/ng.3914 28714974 PMC5577383

[24] Nelson CP, Goel A, Butterworth AS, Kanoni S, Webb TR, Marouli E, et al. Association analyses based on false discovery rate implicate new loci for coronary artery disease. Nat Genet. 2017;49:1385–91. doi: 10.1038/ng.3913 28714975

[25] Allen F, Crepaldi L, Alsinet C, Strong AJ, Kleshchevnikov V, De Angeli P, et al. Predicting the mutations generated by repair of Cas9-induced double-strand breaks. Nat Biotechnol. Springer Science and Business Media LLC; 2018;37:64–72. doi: 10.1038/nbt.4317 30480667 PMC6949135

[26] Boix CA, James BT, Park YP, Meuleman W, Kellis M. Regulatory genomic circuitry of human disease loci by integrative epigenomics. Nature. Springer Science and Business Media LLC; 2021;590:300–7. doi: 10.1038/s41586-020-03145-z 33536621 PMC7875769

[27] Canver MC, Smith EC, Sher F, Pinello L, Sanjana NE, Shalem O, et al. BCL11A enhancer dissection by Cas9-mediated in situ saturating mutagenesis. Nature. 2015;527:192–7. doi: 10.1038/nature15521 26375006 PMC4644101

[28] Korkmaz G, Lopes R, Ugalde AP, Nevedomskaya E, Han R, Myacheva K, et al. Functional genetic screens for enhancer elements in the human genome using CRISPR-Cas9. Nat Biotechnol. 2016;34:192–8. doi: 10.1038/nbt.3450 26751173

[29] Lopes R, Korkmaz G, Agami R. Applying CRISPR-Cas9 tools to identify and characterize transcriptional enhancers. Nat Rev Mol Cell Biol. 2016;17:597–604. doi: 10.1038/nrm.2016.79 27381243

[30] Ioka T, Tasaki H, Yashiro A, Yamashita K, Ozumi K, Tsutsui M, et al. Association between plasma lipoprotein(a) and endothelial dysfunction in normocholesterolemic and non-diabetic patients with angiographically normal coronary arteries. Circ J. 2002;66:267–71. doi: 10.1253/circj.66.267 11922276

[31] Tchantchou F, Graves M, Falcone D, Shea TB. S-adenosylmethionine mediates glutathione efficacy by increasing glutathione S-transferase activity: implications for S-adenosyl methionine as a neuroprotective dietary supplement. J Alzheimers Dis. 2008;14:323–8. doi: 10.3233/jad-2008-14306 18599958

[32] Stolze LK, Conklin AC, Whalen MB, López Rodríguez M, Õunap K, Selvarajan I, et al. Systems Genetics in Human Endothelial Cells Identifies Non-coding Variants Modifying Enhancers, Expression, and Complex Disease Traits. Am J Hum Genet [Internet]. 2020; Available from: doi: 10.1016/j.ajhg.2020.04.008 32442411 PMC7273528

[33] Fulco CP, Nasser J, Jones TR, Munson G, Bergman DT, Subramanian V, et al. Activity-by-contact model of enhancer-promoter regulation from thousands of CRISPR perturbations. Nat Genet. 2019;51:1664–9. doi: 10.1038/s41588-019-0538-0 31784727 PMC6886585

[34] Yang X, Yang W, McVey DG, Zhao G, Hu J, Poston RN, et al. FURIN Expression in Vascular Endothelial Cells Is Modulated by a Coronary Artery Disease–Associated Genetic Variant and Influences Monocyte Transendothelial Migration [Internet]. Journal of the American Heart Association. 2020. Available from: 10.1161/jaha.119.014333 PMC7070217 32067586

[35] Soubeyrand S, Lau P, Peters V, McPherson R. Off-target effects of CRISPRa on interleukin-6 expression. PLoS One. 2019;14:e0224113. doi: 10.1371/journal.pone.0224113 31658298 PMC6816553

[36] Wirka RC, Wagh D, Paik DT, Pjanic M, Nguyen T, Miller CL, et al. Atheroprotective roles of smooth muscle cell phenotypic modulation and the TCF21 disease gene as revealed by single-cell analysis. Nat Med. 2019;25:1280–9. doi: 10.1038/s41591-019-0512-5 31359001 PMC7274198

[37] Pu X, Chan K, Yang W, Xiao Q, Zhang L, Moore AD, et al. Effect of a coronary-heart-disease-associated variant of ADAMTS7 on endothelial cell angiogenesis. Atherosclerosis. 2020;296:11–7. doi: 10.1016/j.atherosclerosis.2020.01.015 32005000

[38] Erdmann J, Stark K, Esslinger UB, Rumpf PM, Koesling D, de Wit C, et al. Dysfunctional nitric oxide signalling increases risk of myocardial infarction. Nature. Springer Science and Business Media LLC; 2013;504:432–6. doi: 10.1038/nature12722 24213632

[39] Cona B, Hayashi T, Yamada A, Shimizu N, Yokota N, Nakato R, et al. The splicing factor DHX38/PRP16 is required for ovarian clear cell carcinoma tumorigenesis, as revealed by a CRISPR-Cas9 screen. FEBS Open Bio [Internet]. Wiley; 2021; Available from: 10.1002/2211-5463.13358 PMC8886329 34965029

[40] Ajmal M, Khan MI, Neveling K, Khan YM, Azam M, Waheed NK, et al. A missense mutation in the splicing factor gene DHX38 is associated with early-onset retinitis pigmentosa with macular coloboma. J Med Genet. BMJ; 2014;51:444–8. doi: 10.1136/jmedgenet-2014-102316 24737827

[41] Deschênes M, Chabot B. The emerging role of alternative splicing in senescence and aging. Aging Cell. Wiley; 2017;16:918–33. doi: 10.1111/acel.12646 28703423 PMC5595669

[42] Cullot G, Boutin J, Toutain J, Prat F, Pennamen P, Rooryck C, et al. CRISPR-Cas9 genome editing induces megabase-scale chromosomal truncations. Nat Commun. Springer Science and Business Media LLC; 2019;10:1136. doi: 10.1038/s41467-019-09006-2 30850590 PMC6408493

[43] Qi LS, Larson MH, Gilbert LA, Doudna JA, Weissman JS, Arkin AP, et al. Repurposing CRISPR as an RNA-guided platform for sequence-specific control of gene expression. Cell. Elsevier BV; 2013;152:1173–83. doi: 10.1016/j.cell.2013.02.022 23452860 PMC3664290

[44] Hilton IB, D’Ippolito AM, Vockley CM, Thakore PI, Crawford GE, Reddy TE, et al. Epigenome editing by a CRISPR-Cas9-based acetyltransferase activates genes from promoters and enhancers. Nat Biotechnol. Springer Science and Business Media LLC; 2015;33:510–7. doi: 10.1038/nbt.3199 25849900 PMC4430400

[45] Goligorsky MS, Chen J, Patschan S. Stress-induced premature senescence of endothelial cells: a perilous state between recovery and point of no return. Curr Opin Hematol. 2009;16:215–9. doi: 10.1097/MOH.0b013e32832a07bd 19318942

[46] Vasile E, Tomita Y, Brown LF, Kocher O, Dvorak HF. Differential expression of thymosin beta-10 by early passage and senescent vascular endothelium is modulated by VPF/VEGF: evidence for senescent endothelial cells in vivo at sites of atherosclerosis. FASEB J. 2001;15:458–66. doi: 10.1096/fj.00-0051com 11156961

[47] Tohru Minamino, Hideaki Miyauchi, Toshihiko Yoshida, Yasuo Ishida, Hideo Yoshida, Issei Komuro. Endothelial Cell Senescence in Human Atherosclerosis. Circulation. American Heart Association; 2002;105:1541–4. 10.1161/01.cir.0000013836.85741.17 11927518

[48] Childs BG, Baker DJ, Wijshake T, Conover CA, Campisi J, van Deursen JM. Senescent intimal foam cells are deleterious at all stages of atherosclerosis. Science. 2016;354:472–7. doi: 10.1126/science.aaf6659 27789842 PMC5112585

[49] Regnault V, Challande P, Pinet F, Li Z, Lacolley P. Cell senescence: basic mechanisms and the need for computational networks in vascular ageing. Cardiovasc Res [Internet]. 2020; Available from: 10.1093/cvr/cvaa318 33206947

[50] Lessard S, Francioli L, Alfoldi J, Tardif J-C, Ellinor PT, MacArthur DG, et al. Human genetic variation alters CRISPR-Cas9 on- and off-targeting specificity at therapeutically implicated loci. Proc Natl Acad Sci U S A. 2017;114:E11257–66. doi: 10.1073/pnas.1714640114 29229813 PMC5748207

[51] Hart T, Brown KR, Sircoulomb F, Rottapel R, Moffat J. Measuring error rates in genomic perturbation screens: gold standards for human functional genomics. Mol Syst Biol. 2014;10:733. doi: 10.15252/msb.20145216 24987113 PMC4299491

[52] Sanjana NE, Shalem O, Zhang F. Improved vectors and genome-wide libraries for CRISPR screening. Nat Methods. 2014;11:783–4. doi: 10.1038/nmeth.3047 25075903 PMC4486245

[53] Lalonde S, Stone OA, Lessard S, Lavertu A, Desjardins J, Beaudoin M, et al. Frameshift indels introduced by genome editing can lead to in-frame exon skipping. PLoS One. 2017;12:e0178700. doi: 10.1371/journal.pone.0178700 28570605 PMC5453576

[54] Ewels P, Magnusson M, Lundin S, Käller M. MultiQC: summarize analysis results for multiple tools and samples in a single report. Bioinformatics. 2016;32:3047–8. doi: 10.1093/bioinformatics/btw354 27312411 PMC5039924

[55] Li W, Xu H, Xiao T, Cong L, Love MI, Zhang F, et al. MAGeCK enables robust identification of essential genes from genome-scale CRISPR/Cas9 knockout screens. Genome Biol. 2014;15:554. doi: 10.1186/s13059-014-0554-4 25476604 PMC4290824

[56] Li W, Köster J, Xu H, Chen C-H, Xiao T, Liu JS, et al. Quality control, modeling, and visualization of CRISPR screens with MAGeCK-VISPR. Genome Biol. 2015;16:281. doi: 10.1186/s13059-015-0843-6 26673418 PMC4699372

[57] McLaren W, Gil L, Hunt SE, Riat HS, Ritchie GRS, Thormann A, et al. The Ensembl Variant Effect Predictor. Genome Biol. 2016;17:122. doi: 10.1186/s13059-016-0974-4 27268795 PMC4893825

[58] Durinck S, Moreau Y, Kasprzyk A, Davis S, De Moor B, Brazma A, et al. BioMart and Bioconductor: a powerful link between biological databases and microarray data analysis. Bioinformatics. 2005;21:3439–40. doi: 10.1093/bioinformatics/bti525 16082012

[59] Durinck S, Spellman PT, Birney E, Huber W. Mapping identifiers for the integration of genomic datasets with the R/Bioconductor package biomaRt. Nat Protoc. 2009;4:1184–91. doi: 10.1038/nprot.2009.97 19617889 PMC3159387

[60] Bhasin JM, Ting AH. Goldmine integrates information placing genomic ranges into meaningful biological contexts. Nucleic Acids Res. 2016;44:5550–6. doi: 10.1093/nar/gkw477 27257071 PMC4937336

[61] Love MI, Huber W, Anders S. Moderated estimation of fold change and dispersion for RNA-seq data with DESeq2. Genome Biol. 2014;15:550. doi: 10.1186/s13059-014-0550-8 25516281 PMC4302049

[62] McInnes L, Healy J, Melville J. UMAP: Uniform Manifold Approximation and Projection for Dimension Reduction [Internet]. arXiv [stat.ML]. 2018. Available from: http://arxiv.org/abs/1802.03426

[63] Shalem O, Sanjana NE, Hartenian E, Shi X, Scott DA, Mikkelson T, et al. Genome-scale CRISPR-Cas9 knockout screening in human cells. Science. 2014;343:84–7. doi: 10.1126/science.1247005 24336571 PMC4089965

[64] Pellenz S, Phelps M, Tang W, Hovde BT, Sinit RB, Fu W, et al. New human chromosomal safe harbor sites for genome engineering with CRISPR/Cas9, TAL effector and homing endonucleases [Internet]. Cold Spring Harbor Laboratory. 2018 [cited 2021 Feb 11]. p. 396390. Available from: https://www.biorxiv.org/content/10.1101/396390v1

[65] Bustin SA, Benes V, Garson JA, Hellemans J, Huggett J, Kubista M, et al. The MIQE guidelines: minimum information for publication of quantitative real-time PCR experiments. Clin Chem. 2009;55:611–22. doi: 10.1373/clinchem.2008.112797 19246619

[66] Brinkman EK, Chen T, Amendola M, van Steensel B. Easy quantitative assessment of genome editing by sequence trace decomposition. Nucleic Acids Res. 2014;42:e168. doi: 10.1093/nar/gku936 25300484 PMC4267669

[67] Kluesner MG, Nedveck DA, Lahr WS, Garbe JR, Abrahante JE, Webber BR, et al. EditR: A method to quantify base editing from Sanger sequencing. CRISPR j. 2018;1:239–50. doi: 10.1089/crispr.2018.0014 31021262 PMC6694769

[68] Bray NL, Pimentel H, Melsted P, Pachter L. Near-optimal probabilistic RNA-seq quantification. Nat Biotechnol. Nature Research; 2016;34:525–7. 10.1038/nbt.3519 27043002

[69] Soneson C, Love MI, Robinson MD. Differential analyses for RNA-seq: transcript-level estimates improve gene-level inferences. F1000Res. 2015;4:1521. doi: 10.12688/f1000research.7563.2 26925227 PMC4712774

[70] Zhu A, Ibrahim JG, Love MI. Heavy-tailed prior distributions for sequence count data: removing the noise and preserving large differences. Bioinformatics. 2019;35:2084–92. doi: 10.1093/bioinformatics/bty895 30395178 PMC6581436

[71] Liberzon A, Birger C, Thorvaldsdóttir H, Ghandi M, Mesirov JP, Tamayo P. The Molecular Signatures Database (MSigDB) hallmark gene set collection. Cell Syst. 2015;1:417–25. doi: 10.1016/j.cels.2015.12.004 26771021 PMC4707969

[72] Yu G, Wang L-G, Han Y, He Q-Y. clusterProfiler: an R package for comparing biological themes among gene clusters. OMICS. 2012;16:284–7. doi: 10.1089/omi.2011.0118 22455463 PMC3339379

[73] Yang R, Van Etten JL, Dehm SM. Indel detection from DNA and RNA sequencing data with transIndel. BMC Genomics. 2018;19:270. doi: 10.1186/s12864-018-4671-4 29673323 PMC5909256

[74] Hasan MS, Wu X, Zhang L. Uncovering missed indels by leveraging unmapped reads. Sci Rep. 2019;9:11093. 31366961 10.1038/s41598-019-47405-z PMC6668410

[75] Alquicira-Hernandez J, Powell JE. Nebulosa recovers single cell gene expression signals by kernel density estimation. Bioinformatics [Internet]. 2021; Available from: doi: 10.1093/bioinformatics/btab003 33459785

[76] Giambartolomei C, Vukcevic D, Schadt EE, Franke L, Hingorani AD, Wallace C, et al. Bayesian test for colocalisation between pairs of genetic association studies using summary statistics. PLoS Genet. 2014;10:e1004383. doi: 10.1371/journal.pgen.1004383 24830394 PMC4022491

[77] Graham SE, Clarke SL, Wu K-HH, Kanoni S, Zajac GJM, Ramdas S, et al. The power of genetic diversity in genome-wide association studies of lipids. Nature. Springer Science and Business Media LLC; 2021;600:675–9. doi: 10.1038/s41586-021-04064-3 34887591 PMC8730582

