# Supplementary figures and images for: Multimodal CRISPR perturbations of GWAS loci associated with coronary artery disease in vascular endothelial cells
Source: PLoS Genet. 2023 Mar 16;19(3):e1010680. doi: 10.1371/journal.pgen.1010680 (PMC10047545; doi:10.1371/journal.pgen.1010680)

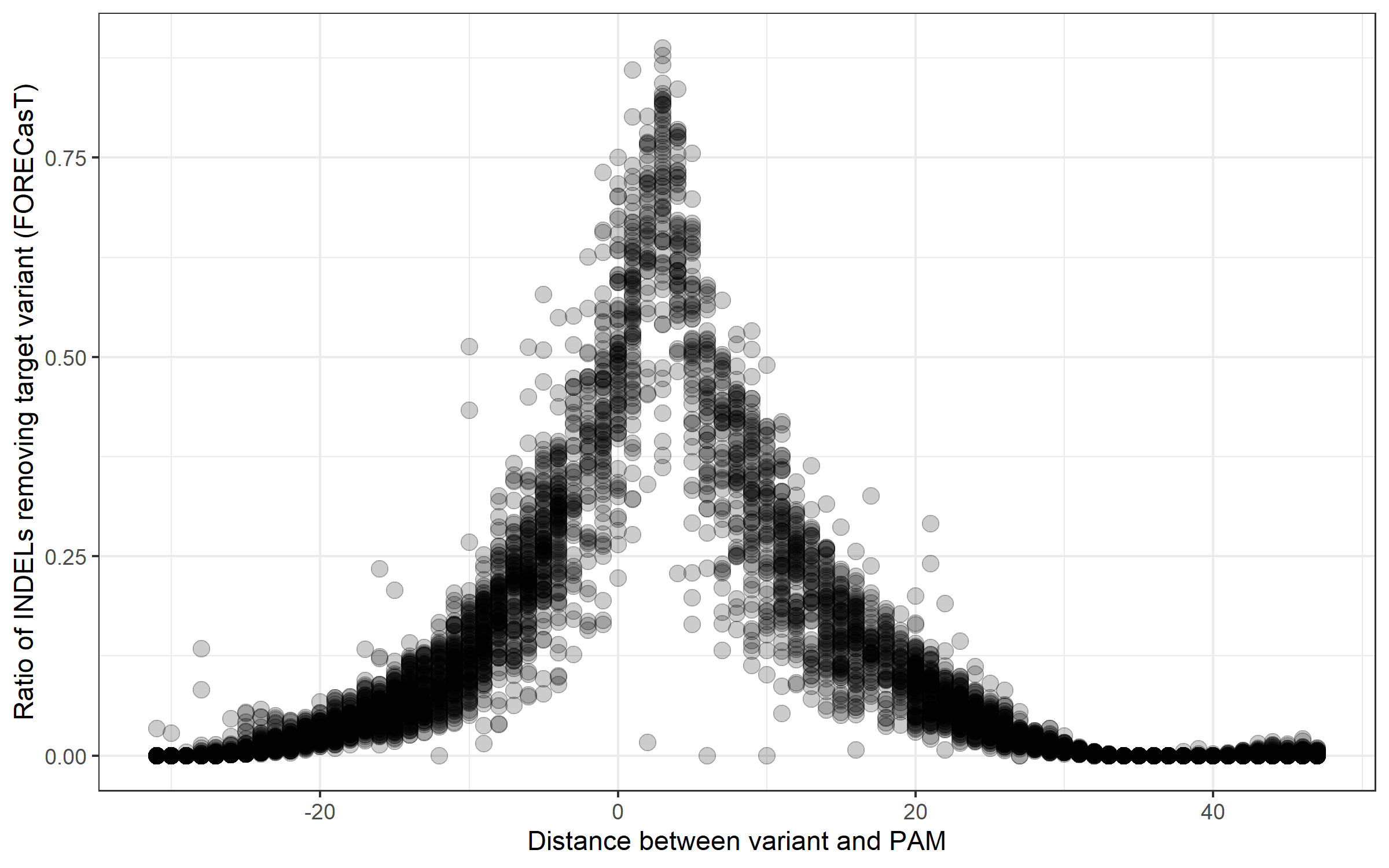

Supplement: S1 Fig — On the x-axis, we report the distance between the CAD variants and the PAM sites. We used the FORECAST algorithm to perform this analysis (Allen et al., Nature Biotech., 2019). (PNG) [file pgen.1010680.s001.png]

## A Cas9: E-Selectin

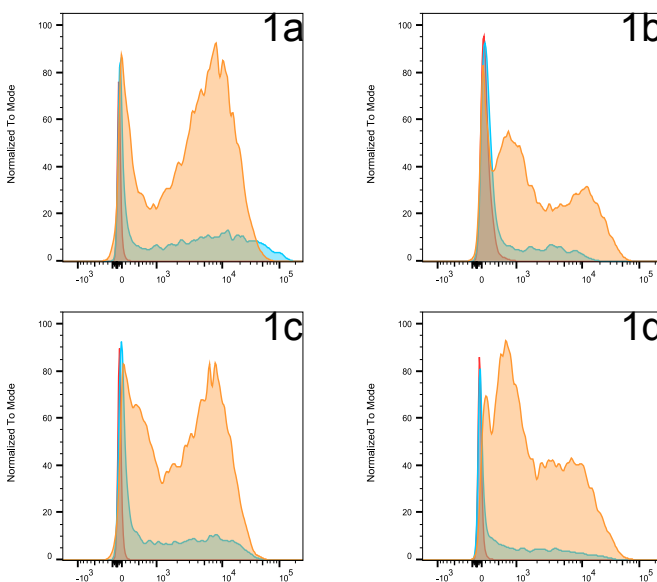

## B Cas9: ICAM-1

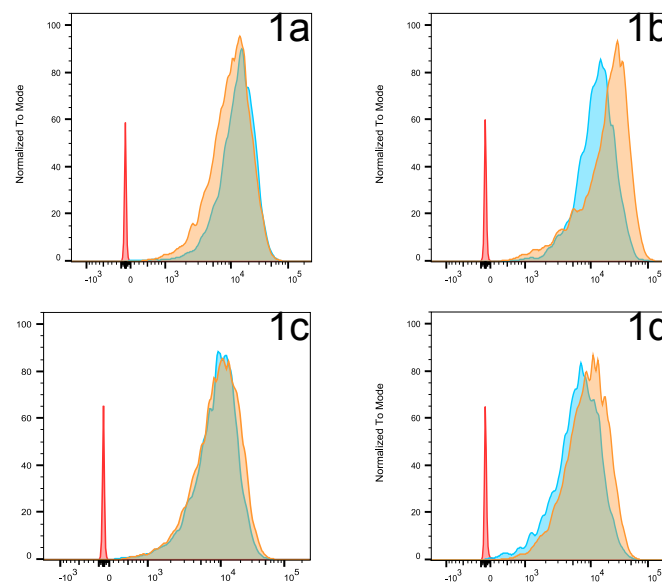

## C Cas9: VCAM-1

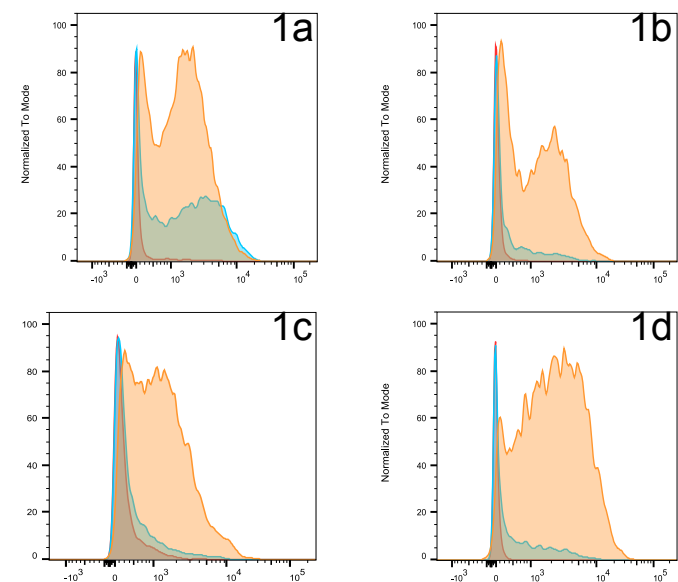

## D Cas9: ROS

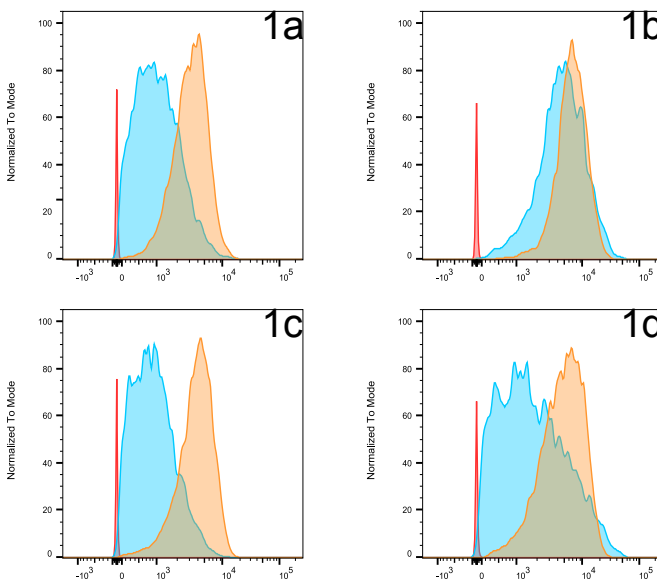

## E Cas9: NO

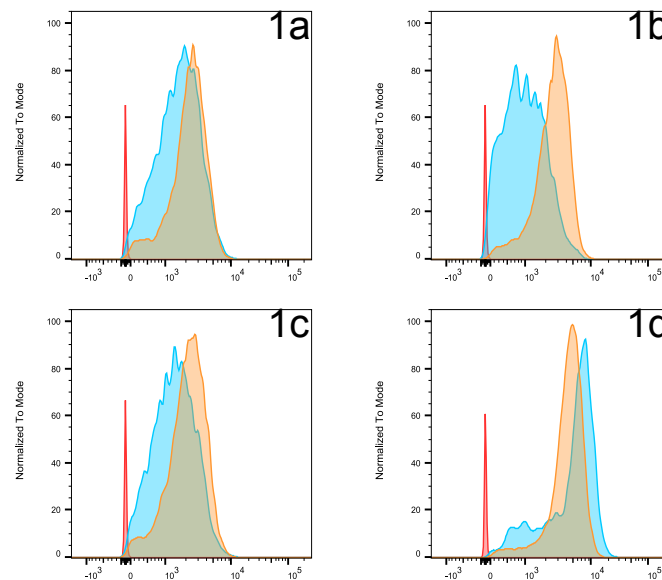

## F Cas9: Ca<sup>2+</sup>

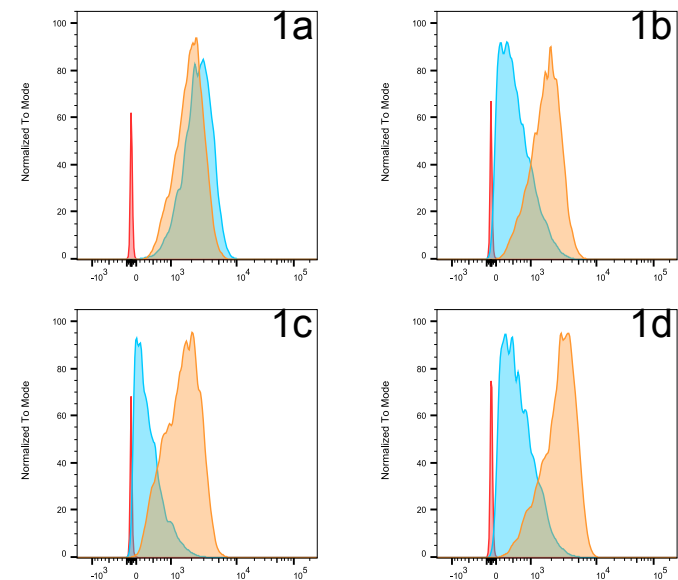

Supplement: S2 Fig — For each experiment, we show a representative figure of the flow cytometry profiles obtained for each of the four lentiviral batches (1a-1d) used in our experiments. Red: Non-infected, non-stained cells; Blue: Non-infected, stained cells; Orange: Infected and stained cells. For E-selectin, ICAM1 and VCAM1, the fluorochrome is PE; for ROS, NO and Ca2+, the fluorochrome is FITC. (PDF) [file pgen.1010680.s002.pdf]

# A CRISPRi: E-Selectin

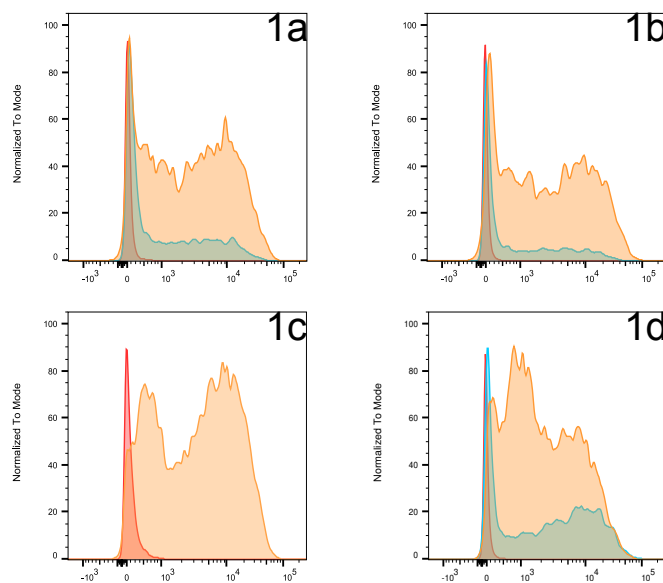

# B CRISPRi: ICAM-1

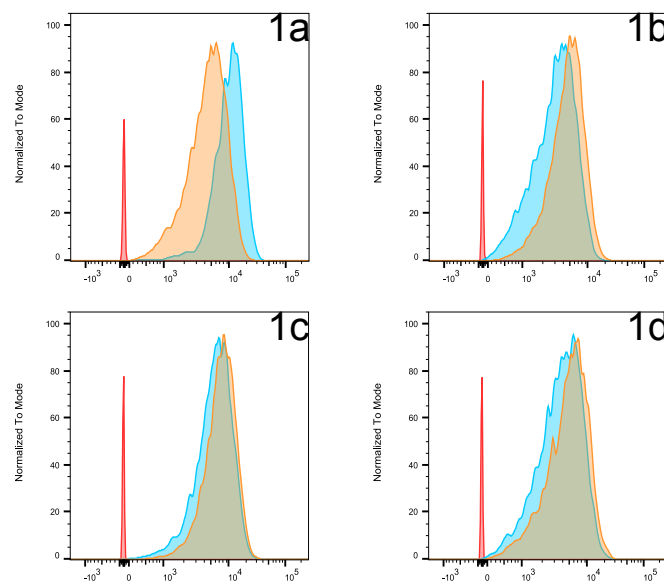

# C CRISPRi: VCAM-1

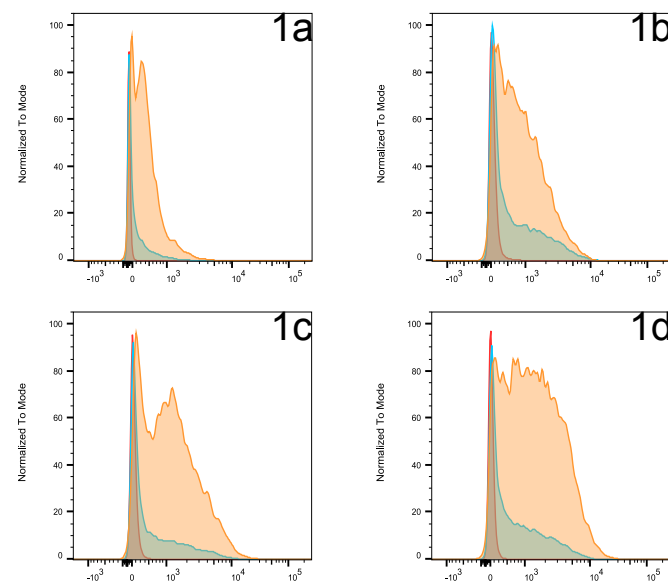

# D CRISPRi: ROS

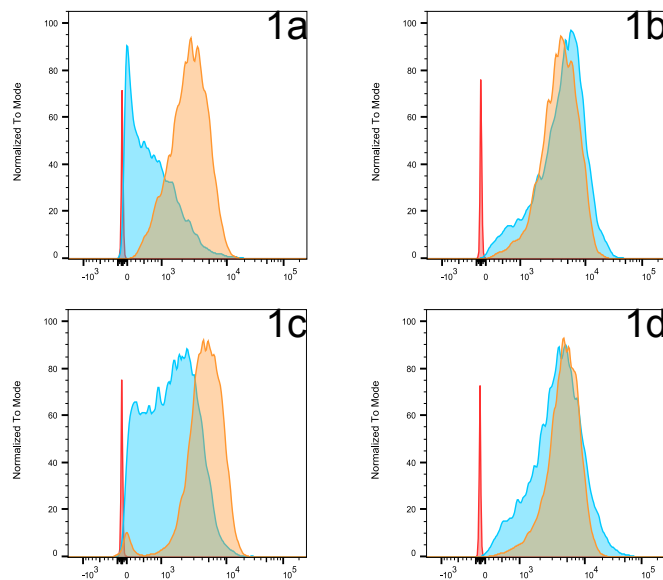

# E CRISPRi: NO

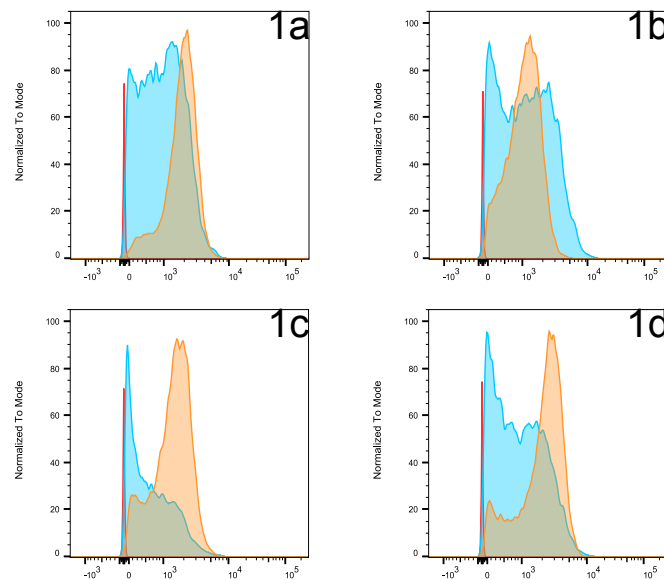

# F CRISPRi: $\text{Ca}^{2+}$

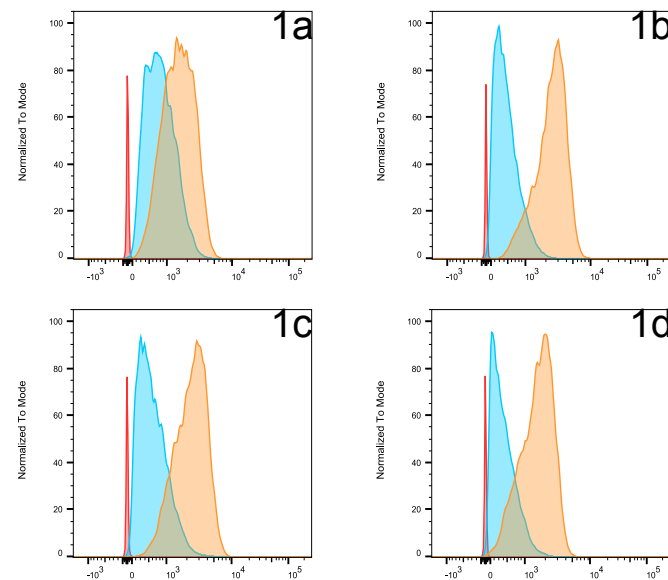

Supplement: S3 Fig — For each experiment, we show a representative figure of the flow cytometry profiles obtained for each of the four lentiviral batches (1a-1d) used in our experiments. Red: Non-infected, non-stained cells; Blue: Non-infected, stained cells; Orange: Infected and stained cells. For E-selectin, ICAM1 and VCAM1, the fluorochrome is PE; for ROS, NO and Ca2+, the fluorochrome is FITC. (PDF) [file pgen.1010680.s003.pdf]

# A CRISPRa: E-Selectin

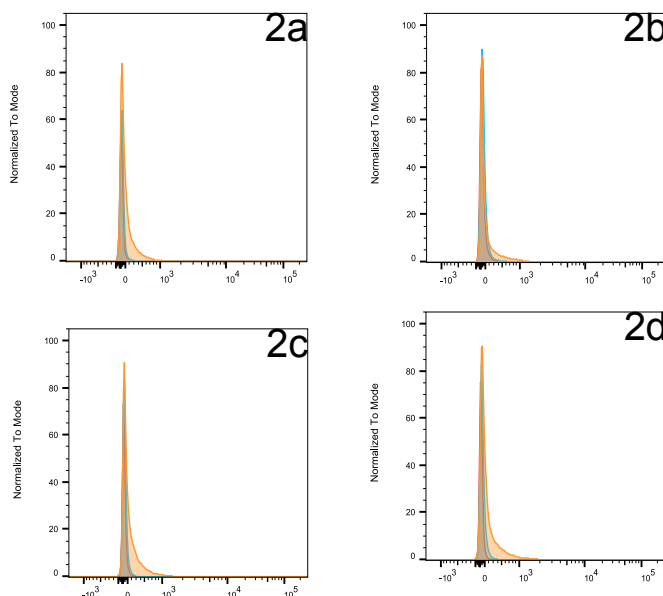

# B CRISPRa: ICAM-1

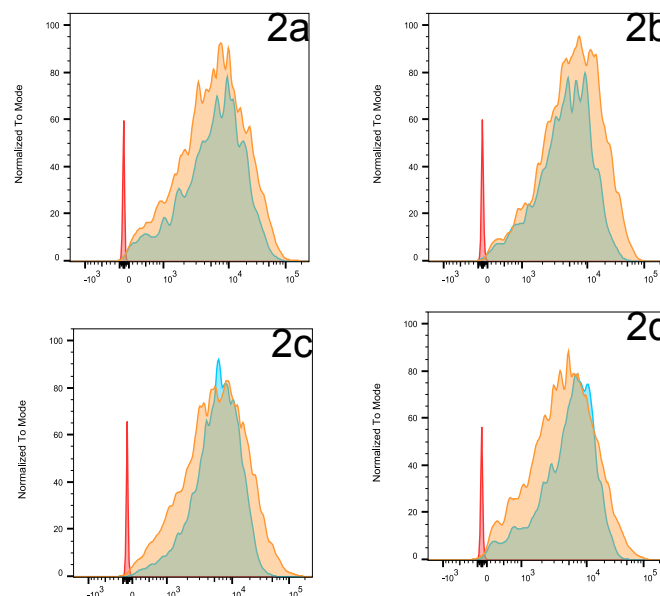

# C CRISPRa: VCAM-1

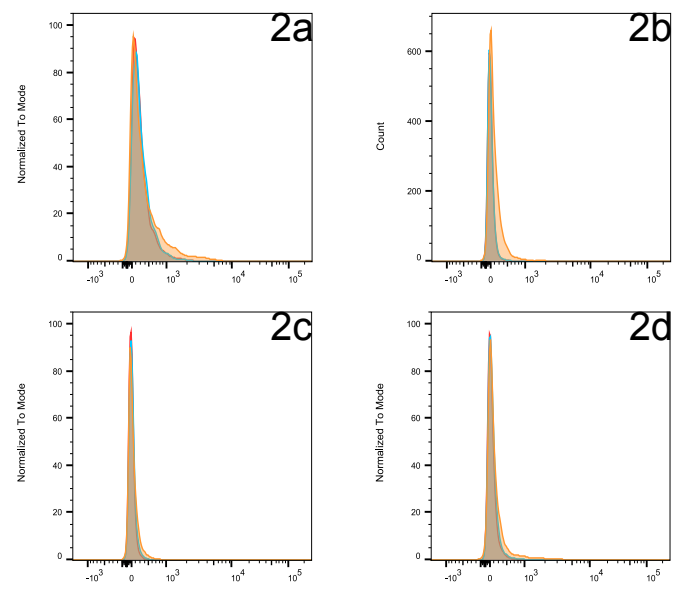

# D CRISPRa: ROS

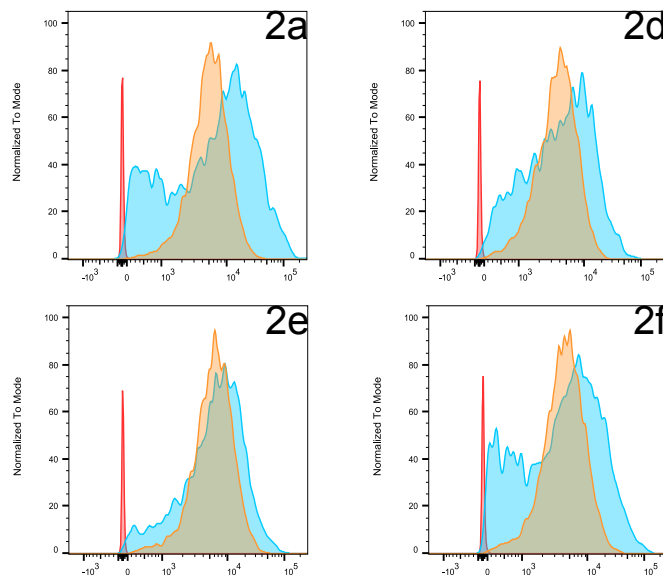

# E CRISPRa: NO

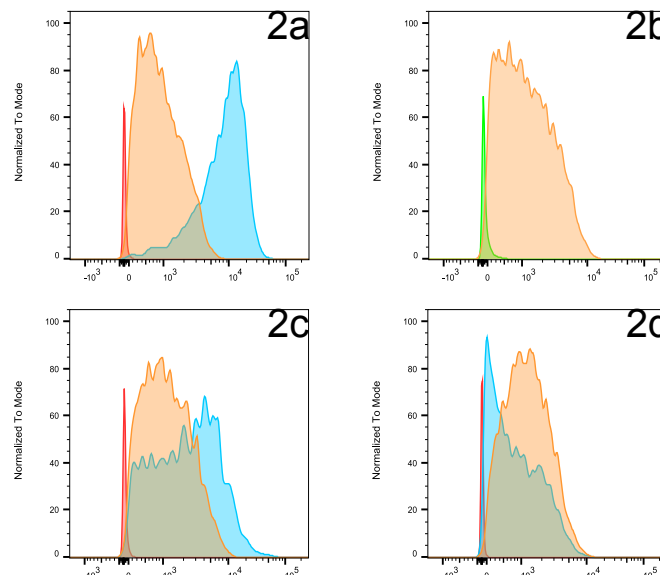

# F CRISPRa: $\text{Ca}^{2+}$

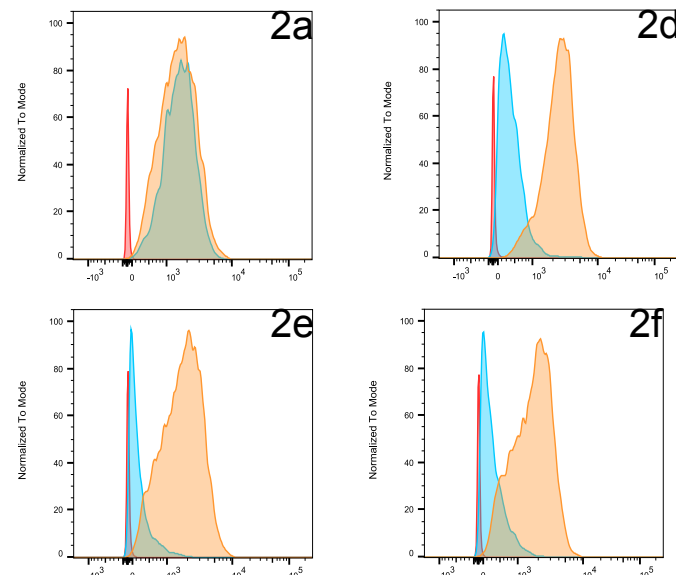

Supplement: S4 Fig — For each experiment, we show a representative figure of the flow cytometry profiles obtained for each of the four lentiviral batches (1a-1d) used in our experiments. Red: Non-infected, non-stained cells; Blue: Non-infected, stained cells; Orange: Infected and stained cells. For E-selectin, ICAM1 and VCAM1, the fluorochrome is PE; for ROS, NO and Ca2+, the fluorochrome is FITC (PDF) [file pgen.1010680.s004.pdf]

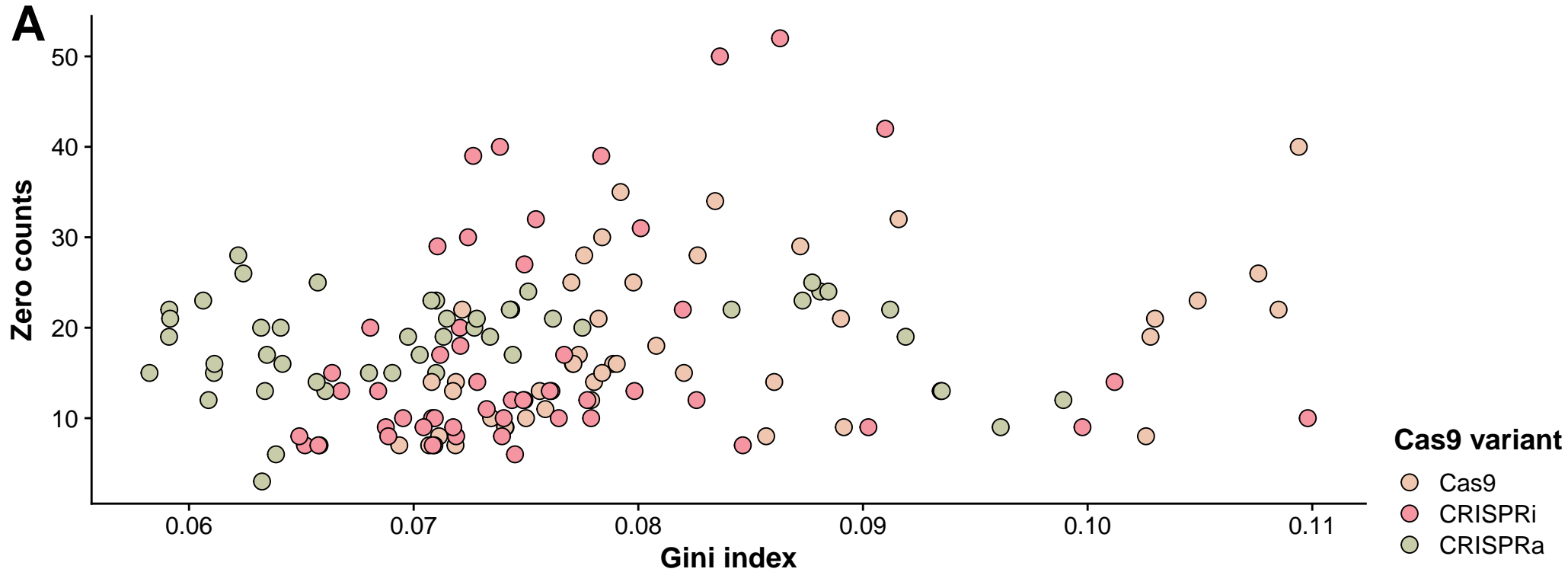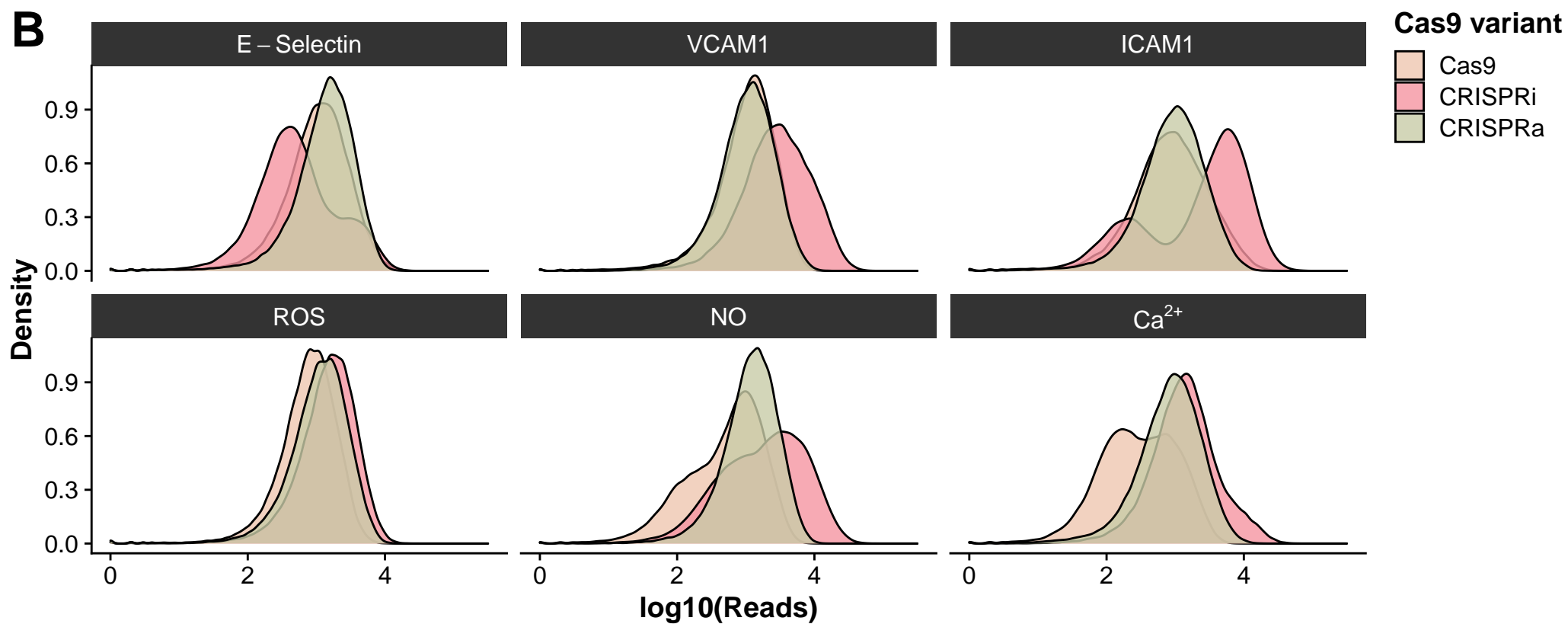

Supplement: S5 Fig — (A) The Gini index is a measure of sgRNA diversity across a sample. A low Gini index indicates that all sgRNAs are equally represented in the sequenced data. The data is stratified (color-coded) by Cas9 proteins. For the sequence data across all experiments, the Gini index is below the recommended threshold (Gini index <0.2,65. The y-axis denotes the number of sgRNAs with 0 reads for each experiment. (B) Density distribution of reads per sgRNA for all endothelial phenotypes and Cas9 proteins (the x-axis is read depth on a log10 scale). (PDF) [file pgen.1010680.s005.pdf]

**A**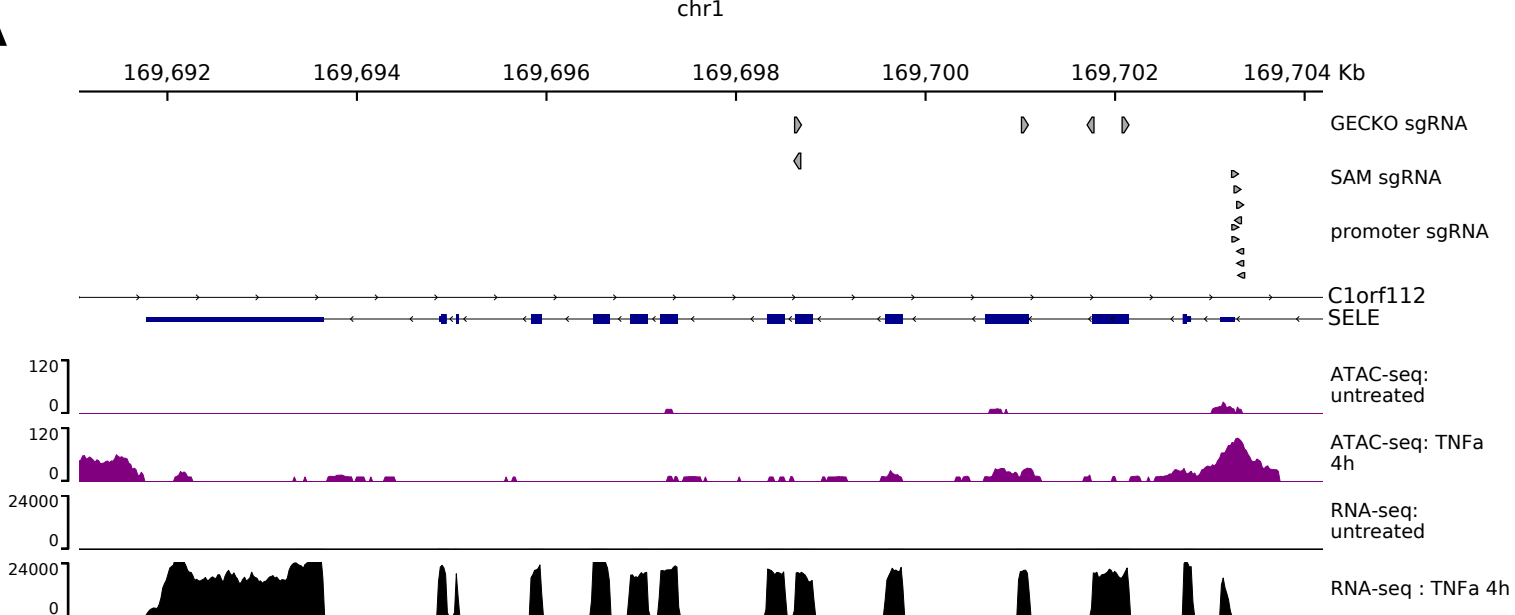**B**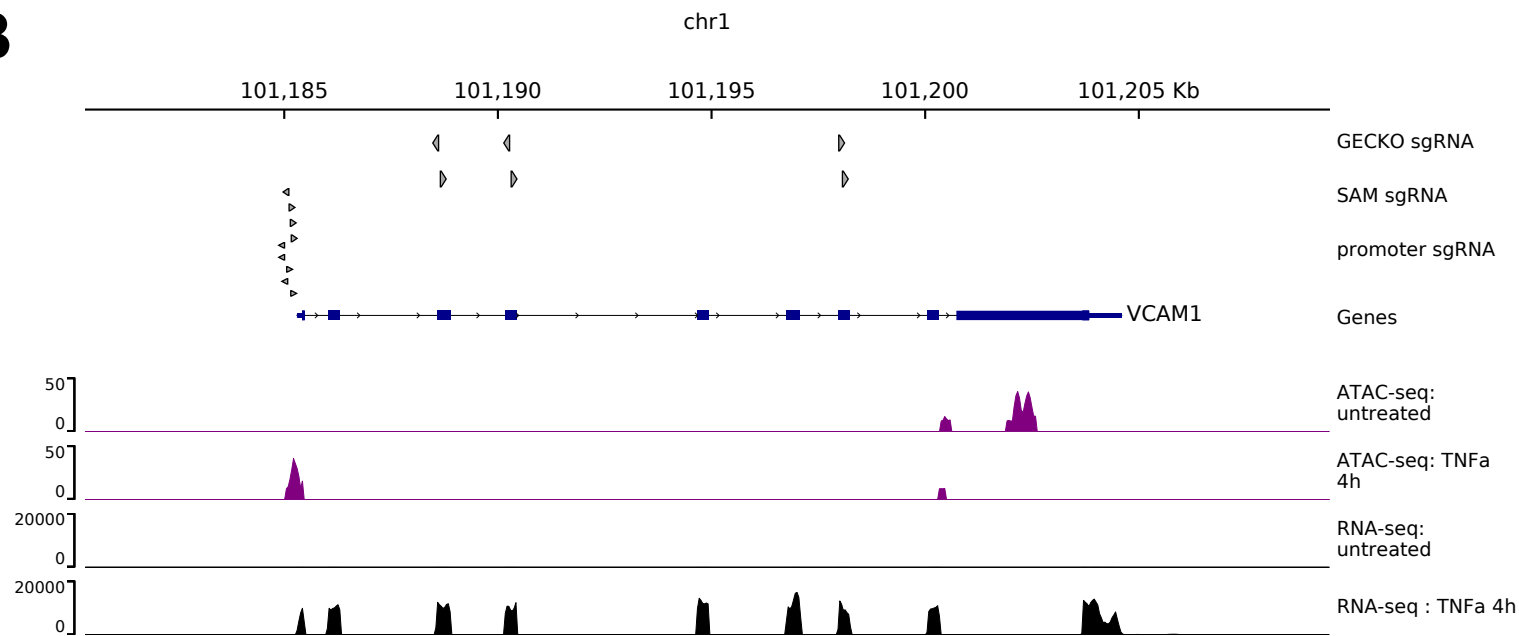**C**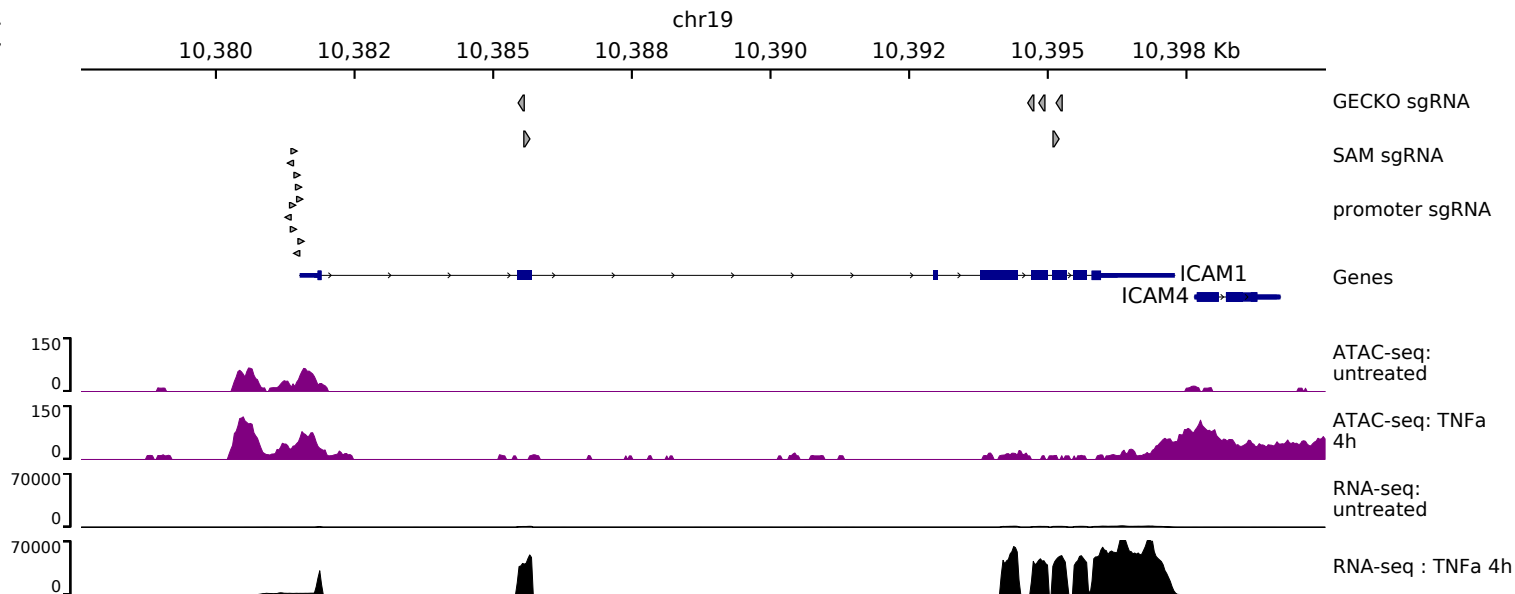

Supplement: S6 Fig — Locus views for adhesion molecule positive control genes (A) SELE (E-selectin), (B) VCAM1 and (C) ICAM1. We also represent the position of the sgRNAs that were tested in the pooled CRISPR screens (GECKO, coding sequences; SAM and promoter, regulatory sequences), as well as RNA-seq and ATAC-seq data in teloHAEC that are un-stimulated or activated for 4 hours with TNFɑ (Lalonde et al., Genome Biol., 2019). (PDF) [file pgen.1010680.s006.pdf]

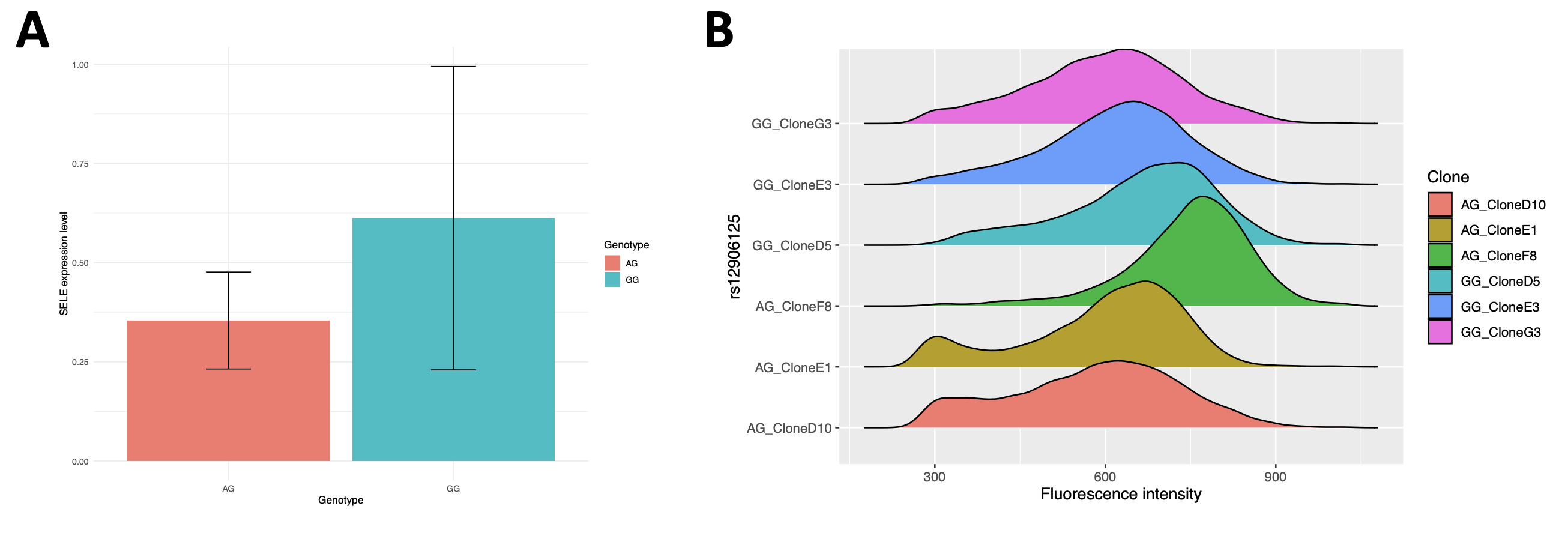

Supplement: S7 Fig — For all experiments, cells were treated for 4 hours with TNFα. (A) SELE mRNA expression levels measured by real-time qPCR. We analyzed at least six clones of each genotype. Based on the CRISPRa (Fig 3B) and FES qPCR experiments (Fig 4C), we expect G/G cells to express SELE at higher levels. The difference is not significant (Student’s t-test P = 0.17). (B) We used flow cytometry to measure E-selectin at the cell membrane of three teloHAEC clones with the A/G genotype and three with the G/G genotype. The difference in mean fluorescence intensity (MFI) between the two groups is non-significant (one-tailed Student’s t-test P = 0.42). (PNG) [file pgen.1010680.s007.png]

A

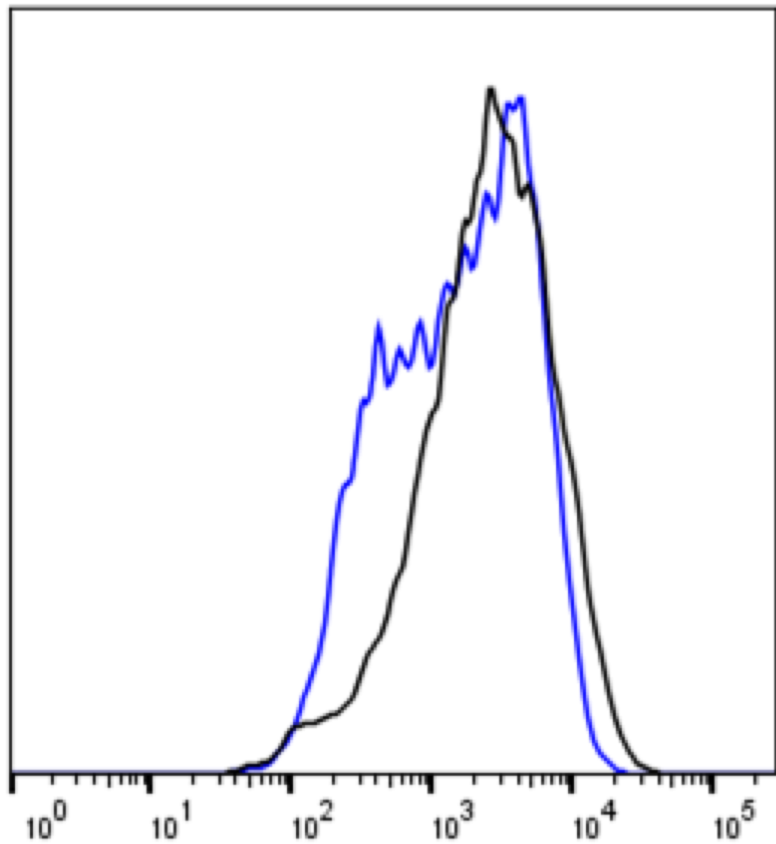

E-selectin fluorescence intensity

B

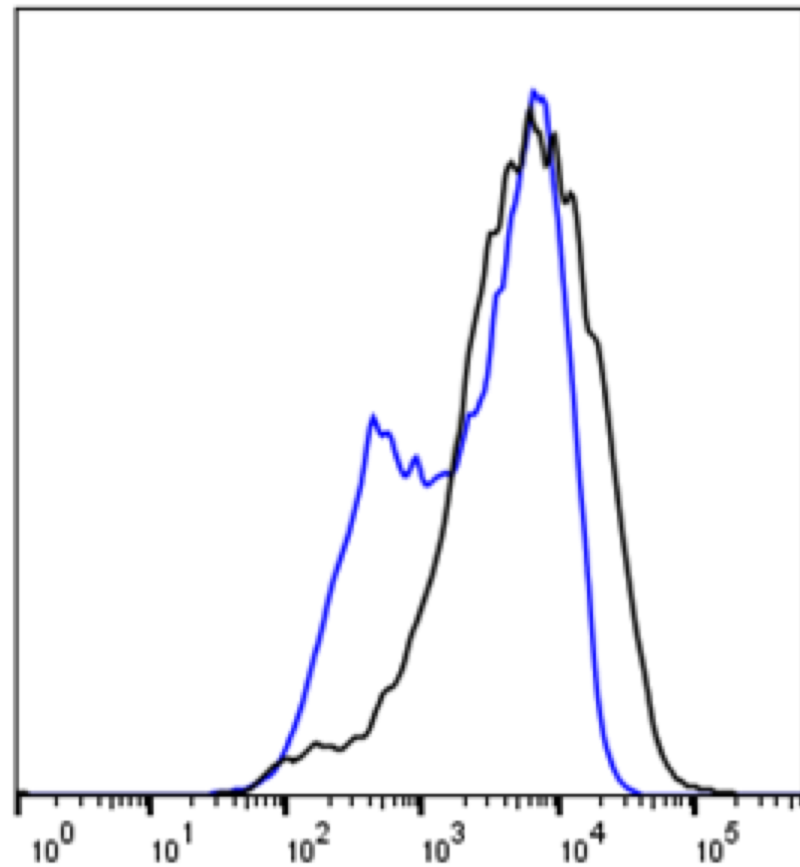

E-selectin fluorescence intensity

Supplement: S8 Fig — Results are shown for two independent biological replicates. At the 10% bottom fraction threshold for E-selectin levels in the control experiments (empty nucleofection, black), we find 24% (panel A) and 31% (panel B) of cells following Cas9 RNP targeting rs2074626 in DHX38. This result confirms that DHX38 inactivation reduces E-selectin presentation at the cell membrane. The fluorochrome is PE. (PDF) [file pgen.1010680.s008.pdf]

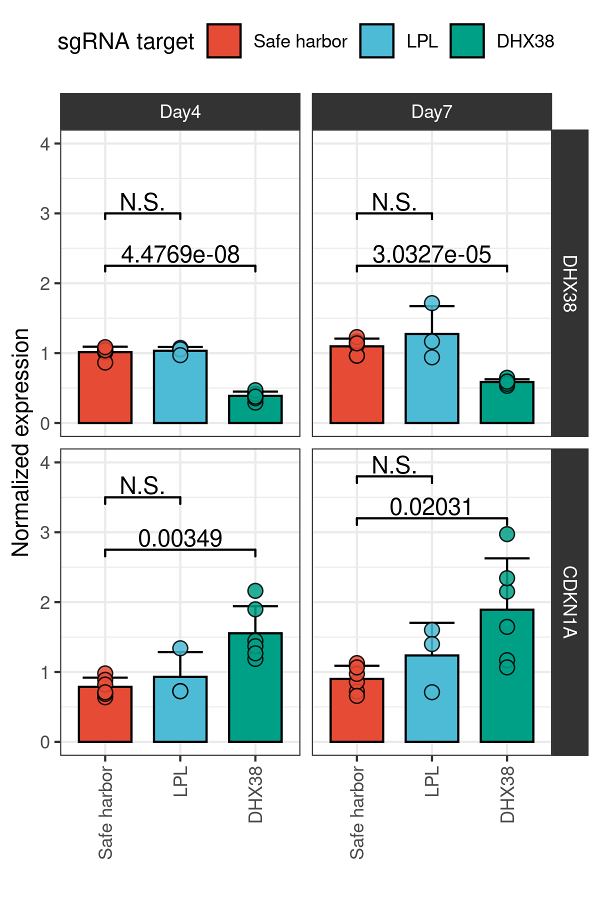

Supplement: S9 Fig — Nucleofection of HCAEC with sgRNAs targeting DHX38 coding exons decrease DHX38 and increase CDKN1A expression levels (4 and 7 days post nucleofection). The sgRNA against LPL was not significant in our pooled CRISPR screen and is used here as a negative control, along with the safe harbor sgRNAs. N.S. = not significant. (PNG) [file pgen.1010680.s009.png]

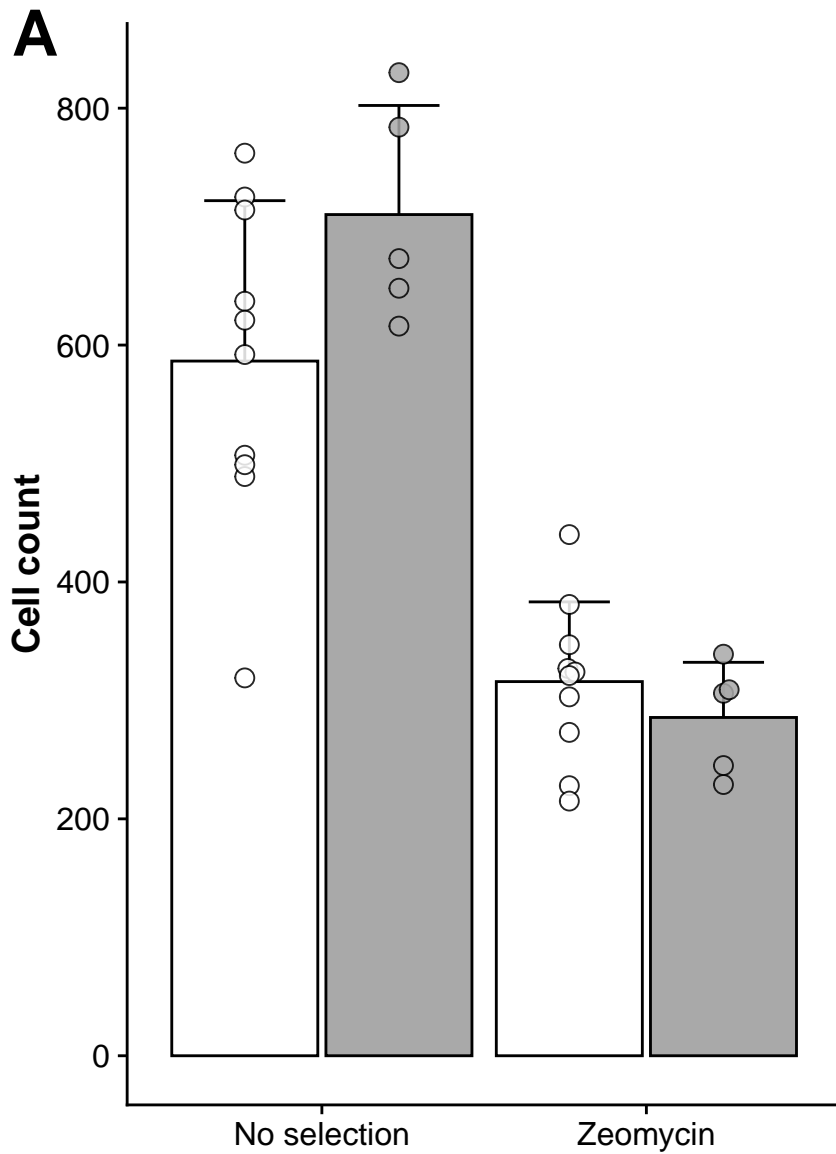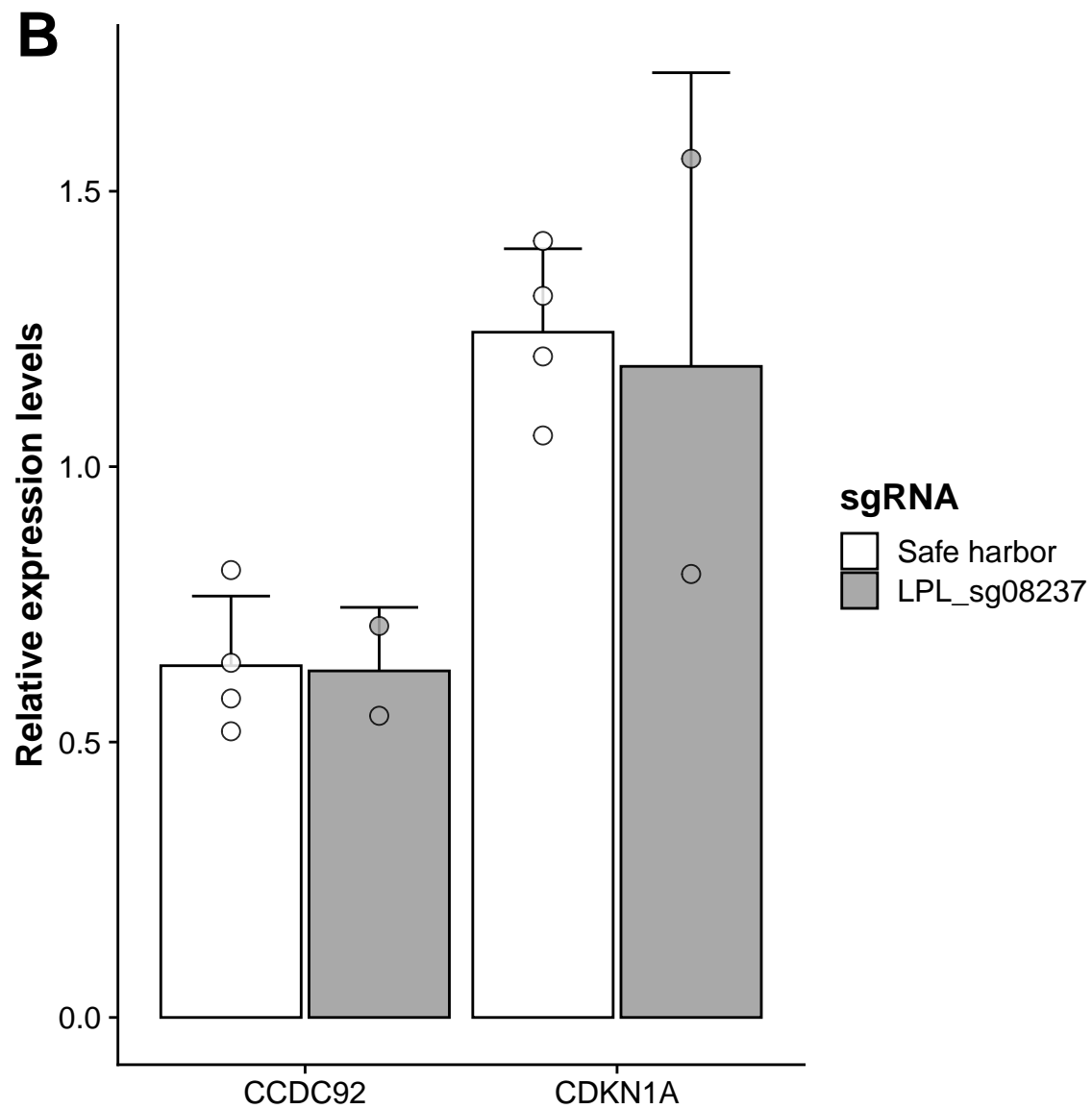

Supplement: S10 Fig — (A) teloHAEC that express dCas9-VP64 were infected with a lentivirus that carries a sgRNA that targets rs1441755 at the LPL locus. This sgRNA was silent in all our pooled CRISPR screens for all six endothelial phenotypes tested. In the absence or presence of antibiotic selection (Zeocin), LPL_sg08237 does not affect cell proliferation. Cell counts are mean ± standard deviation of 10 and 5 images for the safe harbor and LPL_sg08237 sgRNAs, respectively. The differences are not significant (Student’s t-test P>0.06). (B) Expression of CCDC92 and CDKN1A in teloHAEC that express dCas9-VP64 (with zeocin selection). Results are mean ± standard deviation from 4 and 2 experiments for the safe harbor and LPL_sg08237 sgRNAs, respectively. The differences are not significant (Student’s t-test P>0.6). (PDF) [file pgen.1010680.s010.pdf]

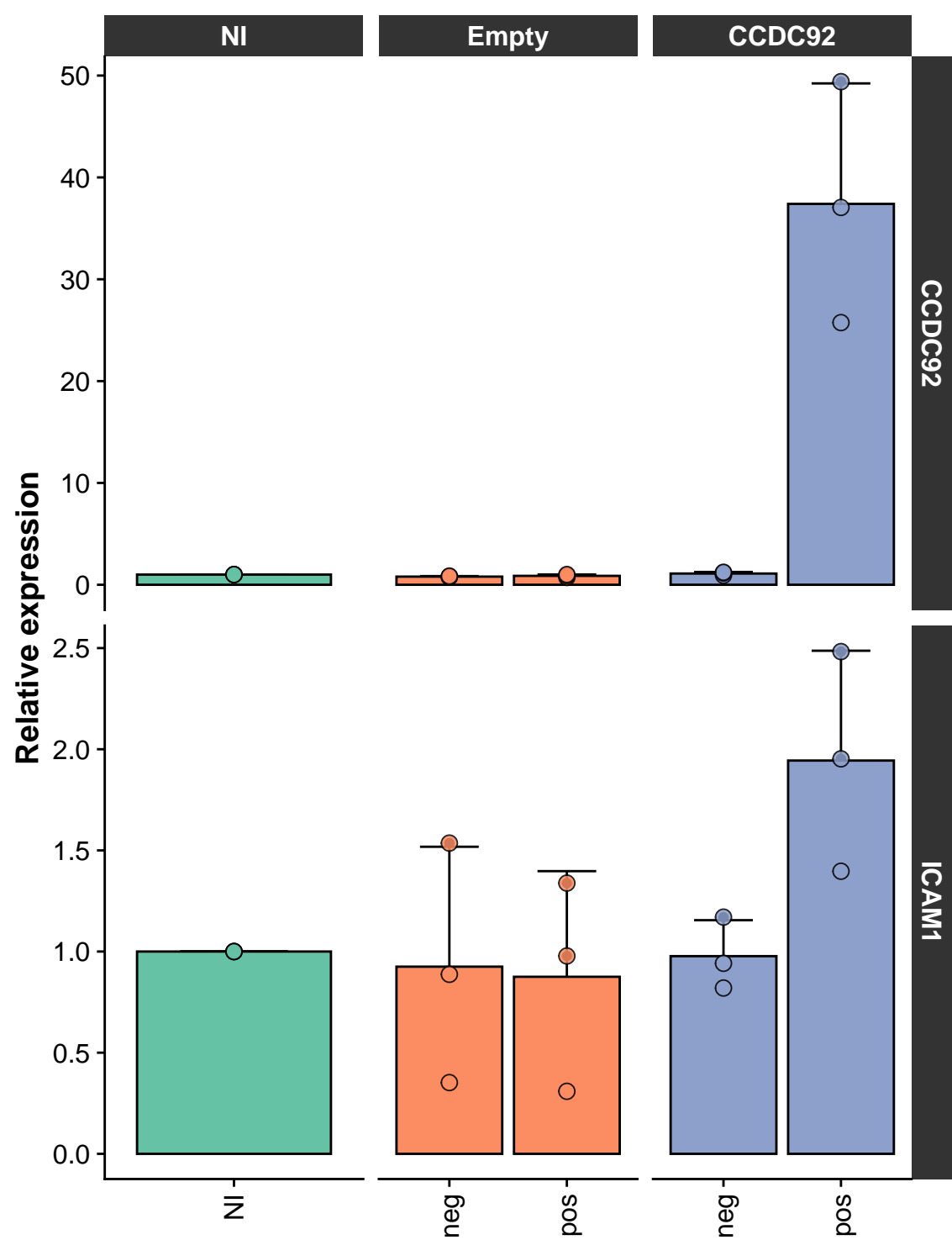

Supplement: S11 Fig — Ectopic over-expression of the CCDC92 ORF via viral infection alongside non-infected (NI) and empty controls (Empty) (top x-axis). For each over-expression experiment, we sorted cells by flow cytometry between mCherry negative (neg) and mCherry positive (pos) cells, and quantify transcripts by quantitative PCR in each fraction individually. Over-expression of the CCDC92 ORF causes a strong induction of CCDC92 expression (pos, top row, blue bars). However, the increase expression in ICAM1 levels is not significant when comparing mCherry positive (i.e. over-expressing CCDC92) and negative cells (bottow row, blue bars). (PDF) [file pgen.1010680.s011.pdf]
